# Catalytic Conversion of Carbon Dioxide through C-N Bond Formation

**DOI:** 10.3390/molecules24010182

**Published:** 2019-01-05

**Authors:** Jing-Yuan Li, Qing-Wen Song, Kan Zhang, Ping Liu

**Affiliations:** State Key Laboratory of Coal Conversion, Institute of Coal Chemistry, Chinese Academy of Sciences, Taiyuan 030001, China; 18334702902@163.com (J.-Y.L.); zhangkan@sxicc.ac.cn (K.Z.); pingliu@sxicc.ac.cn (P.L.)

**Keywords:** carbon dioxide utilization, chemicals, C-N bond formation, hydrogenation, reaction mechanism, synthetic methods

## Abstract

From the viewpoint of green chemistry and sustainable development, it is of great significance to synthesize chemicals from CO_2_ as C_1_ source through C-N bond formation. During the past several decade years, many studies on C-N bond formation reaction were involved, and many efforts have been made on the theory. Nevertheless, several great challenges such as thermodynamic limitation, low catalytic efficiency and selectivity, and high pressure etc. are still suffered. Herein, recent advances are highlighted on the development of catalytic methods for chemical fixation of CO_2_ to various chemicals through C-N bond formation. Meanwhile, the catalytic systems (metal and metal-free catalysis), strategies and catalytic mechanism are summarized and discussed in detail. Besides, this review also covers some novel synthetic strategies to urethanes based on amines and CO_2_. Finally, the regulatory strategies on functionalization of CO_2_ for *N*-methylation/*N*-formylation of amines with phenylsilane and heterogeneous catalysis *N*-methylation of amines with CO_2_ and H_2_ are emphasized.

## 1. Introduction

With the development of green chemistry, sustainable raw materials are urgently required, and growing attention is being paid to green resources such as biomass [1], carbon dioxide (CO_2_) [2,3,4] etc. due to their good features such as abundance, renewability and sustainability. In the past several decades, utilization of CO_2_ as an ideal C_1_ building block become a hot and promising field in both academic and industrial respects. A large number of advantages promoted the wide and deep research on catalytic conversion of CO_2_ into chemicals (C-O, C-N and C-C bond construction) and fuels [5,6,7]. Though great breakthroughs have been achieved during these years, however, CO_2_ conversion still faces many challenges due to its thermodynamic stability and kinetic inertness. Therefore, many strategies have been employed for effective CO_2_ conversion, including CO_2_ and substrate activation and active reagents (such as hydrogen, alkene, alkyne, epoxides, amines, etc.) [8,9,10,11] with high free-energy. Among the active substrates, amines were demonstrated to be some of the most high-efficiency candidates for chemical fixation of CO_2_ through C-N bond formation. The earliest and most successful example is the production of urea from ammonia and CO_2_, which accounts for most (>90%) of all of the industrial chemicals produced using CO_2_ as a chemical feedstock so far. Besides, many other N-contained chemicals such as urea derivatives, carbamates, oxazolinones, quinazoline-2,4(1*H*,3*H*)-diones, formamides, methylamines, etc. could also be synthesized through C-N bond formation from CO_2_ and various amines or their derivatives [9].

In 2011, the He group comprehensively reviewed the development of CO_2_ conversion through C-N bond formation with the production of oxazolidinones, quinazolines, carbamates, isocyanates and polyurethanes, etc. [6] In the subsequent years, several short reviews describing special fields were also reviewed. For example, catalytic strategy insights were gained on the reaction of CO_2_ and aziridines (a 100% atom economical route) to prepare oxazolidinones and polyurethanes in 2012 [12] and 2017 (heterogeneous catalysis schemes) [13]. Subsequently in 2013, Ghosh et al. summarized various synthetic methods to produce oxazolidinones from CO_2_ with a variety of aziridines, propargylamines and 2-aminoalcohols [14]. In addition, part of a mini-review from the Tomishige group focuses on the direct conversion of CO_2_ with aminoalcohols and diamines to give cyclic carbamates and cyclic ureas via heterogeneous catalysts [15]. Recently, Li et al. focused on catalytic synthesis of urea derivatives using CO_2_ and amines [16]. Moreover, a book chapter from the He group mainly focuses on the synthesis of carbonyl-containing chemicals, including 2-oxazolidinones and quinazoline-2,4(1*H*,3*H*)-diones, through C-N bond formation [17].

**Scheme 1:**
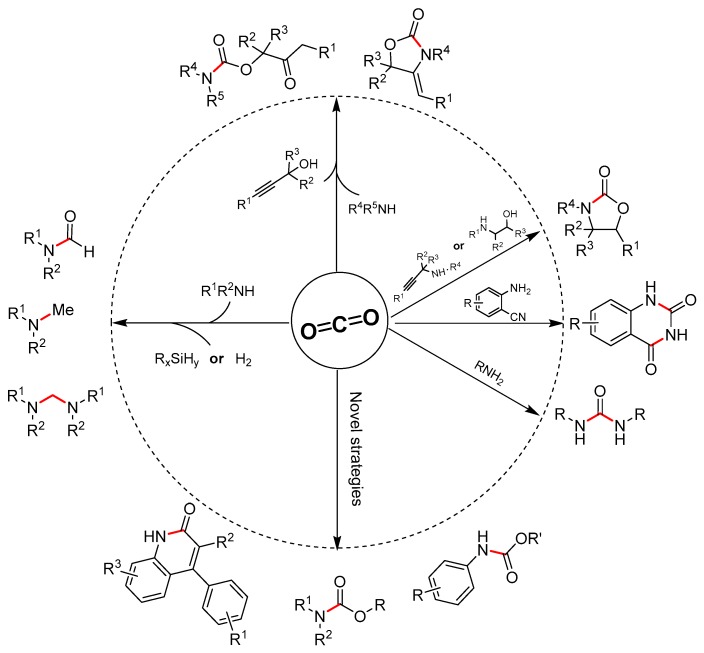
CO_2_ conversion through C-N bond formation reactions described in this work.

Methodology research will provide a solid theory for CO_2_ conversion into value-added products through C-N bond formation, and novel strategies have sprung up in the past few years. Herein, we would like to highlight recent (2014–2018) developments in catalytic methods for chemical fixation of CO_2_ to form various chemicals through C-N bond formation (Scheme 1). Notably, the work described in the previous reviews will not be discussed in this review, unless specially required in individual cases.

## 2. Chemical Fixation of CO_2_ through C-N Bond Formation

### 2.1. Sequential Carboxylation and Cyclization with C-O/C-N Bond Formation

#### 2.1.1. Synthesis of Oxazolidinone Derivatives

Oxazolidinones, a class of five-membered cyclic carbamates, display biological activities and good antibacterial properties. Substituted oxazolidinones are not only used as substrates in organic synthesis, but also as chiral auxiliaries in asymmetric synthesis [18]. In particular, some oxazolidinones can be used as the core unit of antibacterial agents, such as toloxatone, linezolid and tedizolid [19]. Oxazolidinones can be prepared through the intramolecular nucleophilic cyclization of propynyl carbamates as well as chemical fixation of CO_2_ with several different reagents such as aziridines, aminoalcohols and propargylic amines [20]. However, from an availability and economic viability viewpoint, the three-component reaction of propargylic alcohols, primary amines and CO_2_ represents the most attractive method for the synthesis of oxazolidinone derivatives [19].

**Scheme 2:**
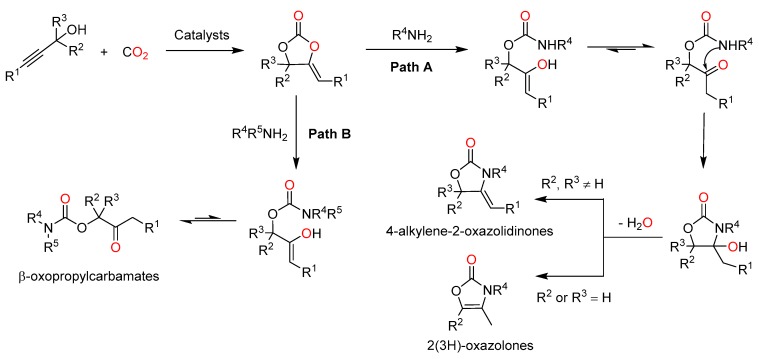
The mechanism for three-component reaction of propargylic alcohols, amines and CO_2._

In the three-component reaction, the carboxylative cyclization of the propargyl alcohol with CO_2_ affords an α-alkylene cyclic carbonate, which then undergoes a nucleophilic attack by a primary amine followed by an intramolecular nucleophilic attack and dehydration to generate 4-alkylene-2-oxazolidinones or 2(3*H*)-oxazolones (See Scheme 2, Path A) [21]. It is worth noting that the formation of products is strongly associated with the substituents on the propargylic alcohols.

**Scheme 3:**
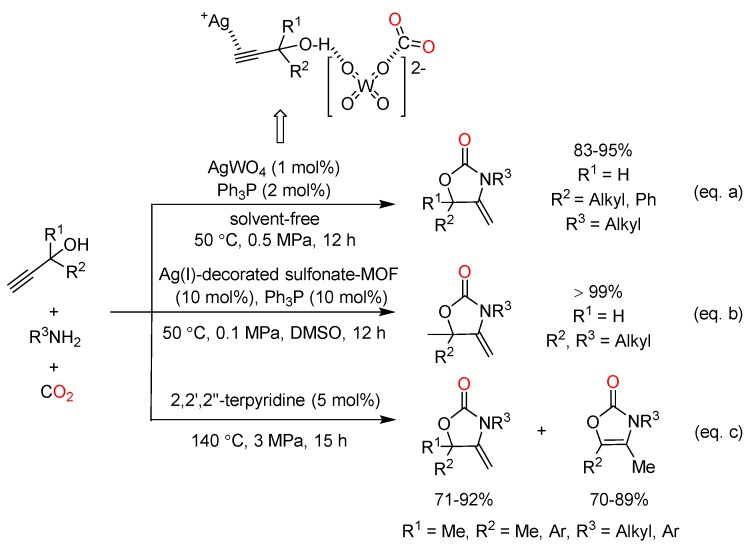
Catalytic systems for the reactions of CO_2_, propargylic alcohols and primary amines.

This reaction was catalyzed by copper salts including CuCl [22] and CuI [23], with the assistance of ionic liquids (ILs) [20] to obtain 4-methylene-2-oxazolidinones or 2(3*H*)-oxazolones. Alternatively, metal-free catalysts, i.e., bicyclic guanidines [24] and [DMIm]BF_4_ [25] are effective in the cycloaddition reactions of CO_2_ with propargylic alcohols and primary amines under relatively mild conditions, although the product yields are not high enough. Moreover, silver salts are suitable catalysts showing high efficiency in the three-component reaction (Scheme 3) [26].

**Figure 1:**
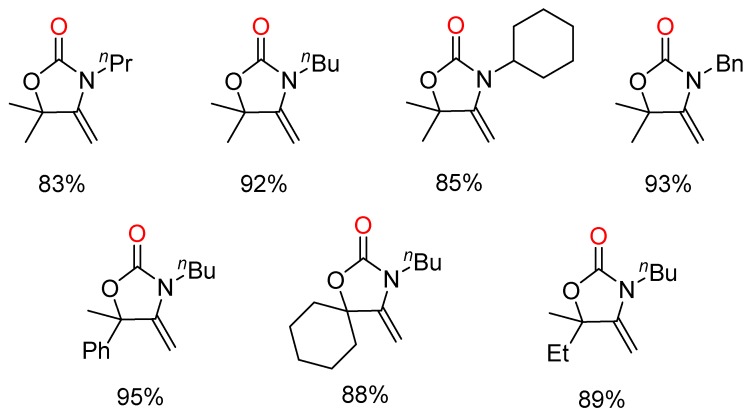
Some 2-oxazolidinone derivatives by Ag_2_WO_4_/Ph_3_P system (isolated yields are given) [21].

Gratifyingly, He et al. obtained corresponding 2-oxazolidinones in moderate to excellent yields by using a bifunctional Ag_2_WO_4_/Ph_3_P system (Scheme 3a and Figure 1) [21]. The reaction was carried out under 0.5 MPa pressure of CO_2_ and 50 °C without using any solvent. As shown in Scheme 3a, the high catalytic activity was ascribed to the dual activation capacity of Ag_2_WO_4_. The silver ion, acting as a π-Lewis acid, could activate the C-C triple bond. Meanwhile, both CO_2_ and the O-H bond of the propargylic alcohol were activated by tungstate anion.

Recently, a noninterpenetrated primitive-cubic (pcu)-type sulfonate-based metal-organic framework (MOF) was synthesized by Fei et al. [27]. Due to the unusual linker-defective nature of the MOF, Ag(I) active sites were incorporated. The synergistic effect of high CO_2_ affinity and alkyne activation nature enabled Ag(I)-decorated sulfonate-MOF successfully catalyze the cyclic carboxylation of propargyl alcohol into oxazolidinones under atmospheric pressure of CO_2_ in the product yield of up to 100% (Scheme 3b). Notably, a fast mass transport resulting from the high surface area and the robust nature of the pcu topology result in a high catalytic efficiency.

An alternative catalyst to obtain 2-oxazolidinone derivatives or 2(3*H*)-oxazolones was a simple organocatalyst, i.e., 2,2′,2′′-terpyridine in the absence of any solvent (Scheme 3c) [18]. Nevertheless, high temperature and CO_2_ pressure was indispensable. Besides, this catalytic system was not suitable for anilines or internal propargyl alcohols.

#### 2.1.2. Synthesis of Carbamates

One of the most important acyclic carbamates, i.e., β-oxopropylcarbamate, has attracted increased attention owing to its growing applications in agriculture and pharmacology [28]. It also acts as a useful intermediate in organic synthesis as well as an amine protective group in peptide chemistry [29]. A one-pot reaction of a propargylic alcohol, secondary amine and CO_2_ is an environmentally benign strategy to produce β-oxopropylcarbamate with high atom economy and easy handling [19]. Unlike the formation of 2-oxazolidinone derivatives, β-oxopropylcarbamates can be obtained via nucleophilic ring-opening of α-alkylidene cyclic carbonates and subsequent tautomerization (See Scheme 2, Path B) [21].

There are various well-established protocols with metal complexes including ruthenium(II) [30], iron complexes based on 1,1′-bis(diphenylphosphino)-ferrocene [31] and silver complexes [29] etc. that were developed before 2012. Additionally, bicyclic guanidines [24] and noncatalytic systems [28] were also demonstrated effective in the coupling of propargylic alcohols, secondary amines and CO_2_. Unfortunately, these approaches generally have severe limitations such as high pressure (≥2 MPa) and additional energy requirements.

In recent years, silver-containing catalytic systems have showed excellent performance in the production of β-oxopropylcarbamates through the three-component reactions, as shown in Table 1. 

**Figure 2:**
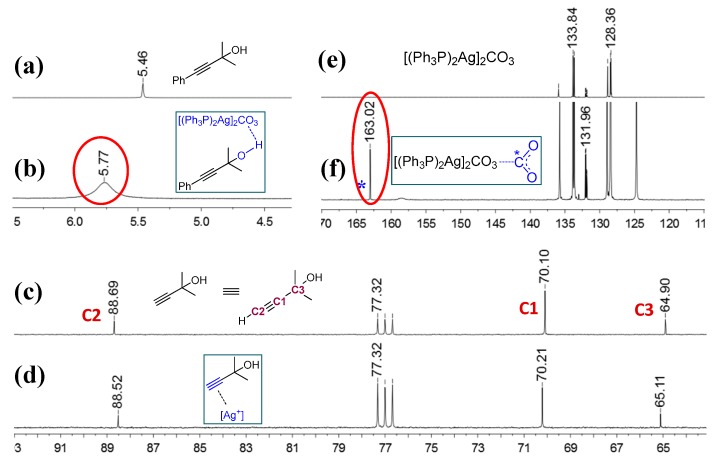
^1^H-NMR (**a**,**b**) and ^13^C-NMR (**c**–**f**) investigation. (**a**,**b**) 2-Methyl-4-phenylbut-3-yn-2-ol (8.0 mg), Ag_2_CO_3_ (2.8 mg) and Ph_3_P (10.5 mg) ([D_6_] DMSO 0.6 mL). (**c**,**d**) 2-methylbut-3-yn-2-ol (20.2 mg), AgNO_3_ (40.8 mg) (CDCl_3_ 0.6 mL). (**e**) [(Ph_3_P)_2_Ag]_2_CO_3_ (158.6 mg) in 0.6 mL of CDCl_3_. (**f**) [(Ph_3_P)_2_Ag]_2_CO_3_ (158.6 mg) in 0.6 mL of CDCl_3_ in the presence of ^13^CO_2_ (0.1 MPa). Reprinted with permission from [32] (Copyright 2014 John Wiley and Sons).

**Figure 3:**
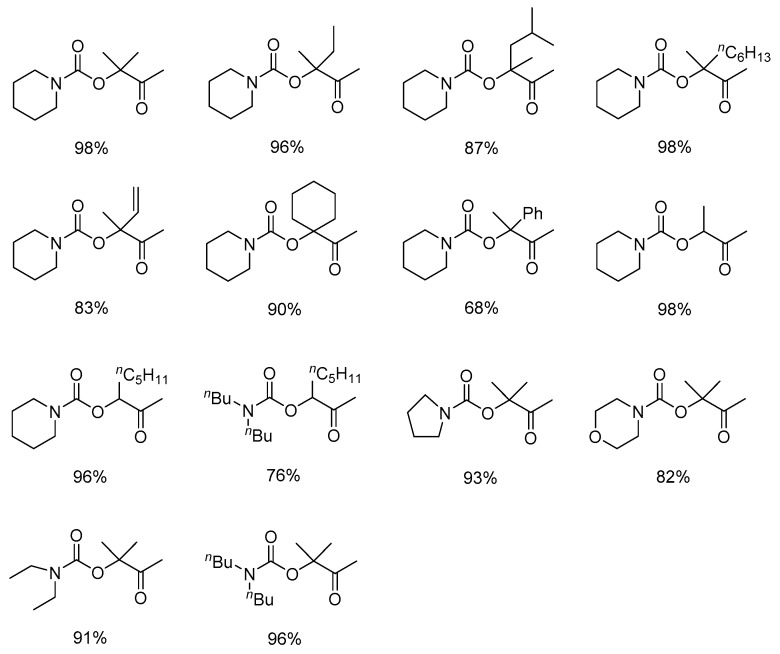
The scope of the [(PPh_3_)_2_Ag]_2_CO_3_ reaction system (isolated yields are given). Reprinted with permission from [32] (Copyright 2014 John Wiley and Sons).

**Scheme 4:**
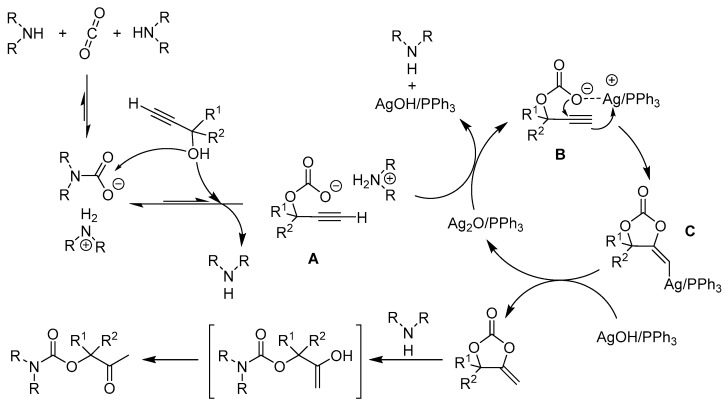
A plausible mechanism for propargylic alcohols with ammonium carbamates [33].

In 2014, He and co-workers reported that silver complex [(PPh_3_)_2_Ag]_2_CO_3_, which could be formed in situ from Ag_2_CO_3_ and PPh_3_, promoted the reaction even under atmospheric CO_2_ pressure [32]. According to the results of ^1^H and ^13^C nuclear magnetic resonance (NMR) spectroscopy, CO_2_ and propargylic alcohol were presumably simultaneously activated by [(PPh_3_)_2_Ag]_2_CO_3_, which greatly facilitated the reaction (Figure 2). Additionally, the scope of the reaction system was demonstrated to be wide (Figure 3). Furthermore, the combinations of Ag_2_WO_4_ and Ag_2_O with PPh_3_, respectively, were also used as catalysts to synthetize β-oxopropylcarbamates under mild reaction conditions. Ag_2_WO_4_/PPh_3_ showed good catalytic activity under the solvent-free condition primarily because of the reactant activation through [Ag(PPh_3_)]^+^ and [WO_4_]^2−^ (Scheme 3a) [21]. Nevertheless, the aromatic amines could not afford the desired products in this system. In contrast, the employment of Ag_2_O/PPh_3_ successfully accomplished the reaction of propargylic alcohols and ammonium carbamate (amine-CO_2_) to achieve the quantitative fixation of CO_2_ [33]. Herein, ammonium carbamate formed from amine and CO_2_ was used as a CO_2_ surrogate. As shown in Scheme 4, propargylic carbonate intermediate **A** is initially generated from the reaction of propargylic alcohol with ammonium carbamate. Subsequently, the intermediate **A** is converted into silver propargylic carbonate **B** in presence of silver(I) catalyst. Then, after the activation of C≡C bond by the silver species, intermediate **C** is obtained and follows by the formation of α-alkylidene cyclic carbonate. Finally, in the presence of a secondary amine, the corresponding β-oxopropylcarbamate can be obtained via tautomerization.

In addition, Song et al. also reported that Ag_2_CO_3_/(*p*-MeOC_6_H_4_)_3_P could catalyze the coupling reaction of terminal propargylic alcohols, CO_2_, and secondary amines with a high turnover number (turnover frequency) of up to 3350 (35 h^−1^) only using 0.01 mol% silver catalyst [34]. More recently, ^13^C_carbonyl_-labeled reactions, ^13^C-NMR spectrum and high-resolution mass spectrometer (HRMS) characterization were performed by Zhang and Hao et al. in the AgCl/Et_4_NCl system. The results further confirmed that carbon atom in -N-C- skeleton of product derived from CO_2_ and the evidence of the previously reported reaction routes [35].

### 2.2. Direct Carboxylation/Cyclization with C-N Bond Formation

#### 2.2.1. Carboxylative Cyclization of Propargyl Amines with Carbon Dioxide

**Scheme 5:**
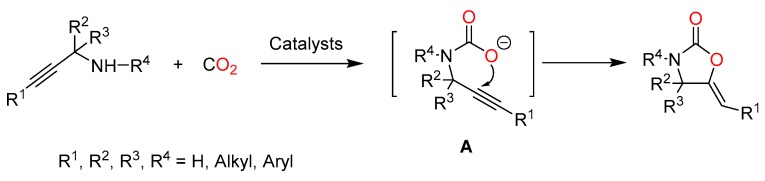
The mechanism for carboxylative cyclization of propargylic amines with CO_2._

Propargylic amines, as some of the important propargylic compounds, are not only easily available but also suitable for further transformations because of their two functional sites (an amino group and a triple bond) [36]. To date, great efforts have been made to develop the carboxylative cyclization of propargylic amines with CO_2_ to obtain 2-oxazolidinones. As described in Scheme 5, CO_2_ initially reacts with propargylic amine to afford intermediate **A**. Then, an intramolecular cyclization occurs with the generation of product. Notably, the second step is considered to be the rate-determining step [37].

**Scheme 6:**
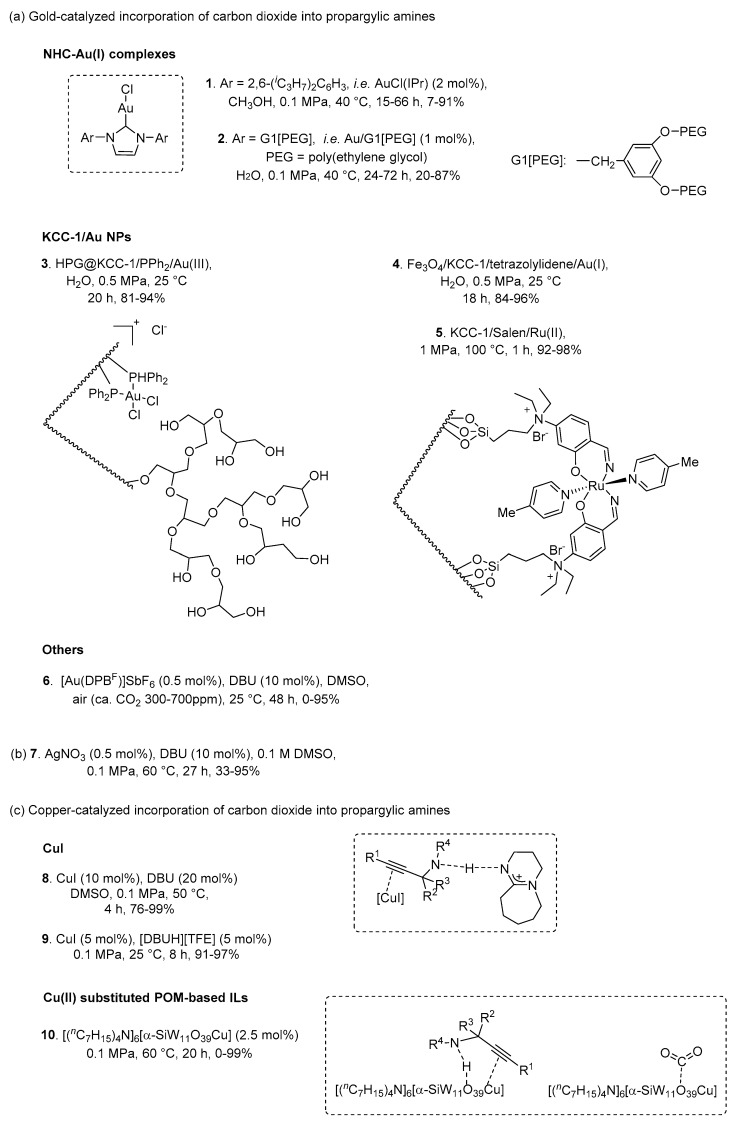
A summary of coinage metals catalysts in carboxylative cyclization of propargylic amines with CO_2._

Taking into account that activation of the carbon-carbon triple bond is of importance to promote the carboxylative cyclization, coinage metals (Au, Ag and Cu) have been extensively employed as catalysts due to π-coordination with the alkyne (Scheme 6). Among them, *N*-heterocyclic carbene (NHC)-Au complexes obviously paved the way to chemical fixation of CO_2_ (**1** and **2**, Scheme 6a). Originally, Ikariya et al. found that AuCl(IPr) (**1**) was capable of catalyzing the synthesis of (Z)-5-alkylidene-1,3-oxazolidin-2-ones in methanol under mild conditions [38]. Further investigation of the substrate scope revealed that the catalytic activity was weakened by the introduction of aromatic substituents at the alkyne terminus [39]. Metallodendrimers have a kind of functional or catalytic site at the core, and their solubility and physical properties can be modified via peripheral structures [40]. Accordingly, amphiphilic dendritic Au/G1[PEG] (**2**) also exhibited good catalytic activity in aqueous media under atmospheric pressure of CO_2_ [41].

Moreover, Sadeghzadeh developed various fibrous nanosilica (KCC-1)/metal nanoparticles (NPs) to catalyze the incorporation of CO_2_ into propargylic amines (**3**, **4** and **5**, Scheme 6a). KCC-1 has a unique fibrous morphology bearing with high surface area, tunable pore size and pore volume, controllable particle size, and improved stability. High polyglycerol (HPG)@KCC-1/PPh_2_/Au (**3**) and Fe_3_O_4_/KCC-1/tetrazolylidene/Au (**4**) catalyst not only catalyzed and gave 2-oxazolidinones in good to excellent yields under 0.5 MPa CO_2_ pressure at room temperature with water as solvent, but also could be recovered and reused several times without appreciable loss of activity [42,43]. Especially, the turnover number of the latter system was up to 1200. In addition, KCC-1/Salen/Ru(II) NPs (**5**) was also efficient in production of oxazolidinones, but high reaction temperature was required [44].

Recently, a gold catalyst containing an Z-type ligand composed of a diphosphine-borane (DPB), namely [Au(DPB^F^)]SbF_6_ (**6**, Scheme 6a) for the carboxylation reaction was developed [45]. The desired products were formed in air (ca. CO_2_ 300–700 ppm) at room temperature in the presence of 1,8-diazabicyclo[5.4.0]undec-7-ene (DBU). However, the yields of the vast majority of corresponding products were around 40%.

As shown in Scheme 6b, silver compounds also displayed excellent performance in the carboxylative cyclization. In 2012, Yoshida et al. reported that AgNO_3_/DBU (**7**) catalytic system was used for CO_2_ capture and transformation at 60 °C and 0.1 MPa CO_2_ in dimethyl sulfoxide (DMSO) [46]. During the process, atmospheric CO_2_ was initially trapped by DBU to give DBU-CO_2_ complex subsequently, which could propel the reaction.

Additionally, a simple and low cost CuI/DBU (**8**, Scheme 6c) system was explored to synthesize 2-oxazolidinones through the coupling reaction of propargylic amines and CO_2_ by Wang et al. [47]. The DFT results suggested that CuI and DBU have an excellent synergistic effect in promoting the reactions. DBU could capture and transfer protons; meanwhile, CuI activated the C≡C triple bond of propargylic amine. Similarly, CuI/[DBUH][TFE] (**9**, Scheme 6c) system also afforded 2-oxazolidinones in outstanding yields under mild conditions [48]. Moreover, He and co-workers exploited a series of metal-substituted polyoxometalate (POM)-based ILs including Cu(II), Co(II), Fe(II), Ni(II), Zn(II) and Mn(II) [49]. Among them, [(*^n^*C_7_H_15_)_4_N]_6_[α-SiW_11_O_39_Cu] (**10**, Scheme 6c) displayed higher catalytic activity than that of other metal-substituted POM-based ILs. It was mainly ascribed to simultaneous activation of propargylic amines and CO_2_ by the single-component catalyst. Notably, this procedure featured as environmentally benign and low energy-input manner.

**Scheme 7:**
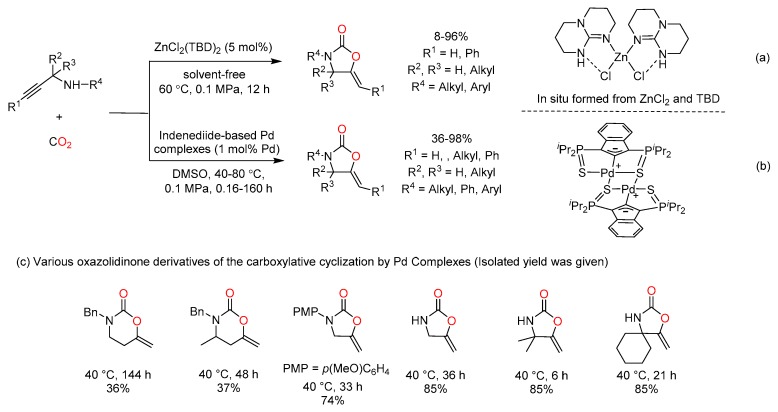
Synthesis of 2-oxazolidinones via Zn- and Pd-catalyzed reaction.

In recent years, considerable attention has also been paid to Zn-based catalysts in CO_2_ fixation because of their tunable Lewis acidity, natural abundance and eco-friendly properties. In 2016, He et al. found that the mononuclear Zn^II^ complex ZnCl_2_(TBD)_2_ (TBD = 1,5,7-triazabicyclo[4.4.0]dec-5-ene) could fix CO_2_ by propargylic amines to deliver corresponding 2-oxazolidinones without any solvent (Scheme 7a) [50]. Nevertheless, the internal propargylic amine was unreactive with CO_2_ in this catalytic system. In contrast, the terminal and internal secondary propargylamines were smoothly transformed into oxazolidinone derivatives by Indenediide-based Pd complex catalysts under mild conditions in DMSO (Scheme 7b) [51]. In particular, six-membered cyclic carbamates and *N*-aryl oxazolidinones were obtained (Scheme 7c). In addition, primary propargylamines, which were prone to occurring side reaction due to the NH_2_ group, also led to the desired oxazolidinones in good yields.

As can be seen from the above examples, the reported metal catalytic systems generally employed expensive noble metals or complicated catalytic components. At the same time, metal-free catalytic processes can reduce the cost and avoid the pollution caused by metals. In recent years, there are well-established protocols with vigorous metal-free catalytic systems such as TBA_2_[WO_4_] (TBA = tetra-*n*-butylammonium) [52], ILs [53], NHCs (e.g., 1,3-di-*t*-butylimidazol-2-ylidene) [54] Bu_4_NF [55,56] and so on.

**Scheme 8:**
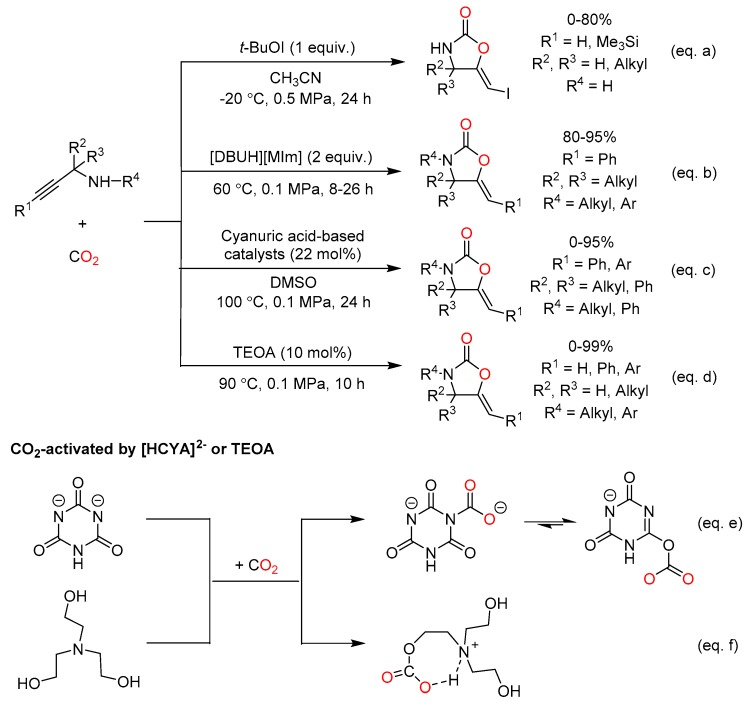
Synthesis of 2-oxazolidinones by metal-free catalytic systems.

In 2012, Minakata reported an effective catalytic system being composed of *t*-BuOI and CH_3_CN for the carboxylative cyclization of propargyl amine and CO_2_, and 2-oxazolidinone bearing iodomethyl group could be generated under mild conditions (Scheme 8a) [57]. Afterwards, with two equivalents of a protic ionic liquid namely 1,8-diazabicyclo-[5.4.0]-7-undecenium 2-methyl- imidazolide ([DBUH][MIm]), the desired products were obtained in good to excellent yields (Scheme 8b) [37]. Gratifyingly, this cheaper and greener catalyst could be easily recycled and reused at least five times. Han et al. also found that [DBUH][MIm] promoted both the CO_2_ electrophilic attack and the intramolecular cyclization step by capturing and transferring proton through theoretical studies, respectively.

Very recently, the reaction of propargylic amines and CO_2_ were carried out in the presence of a cyanuric acid (H_3_CYA)-based organocatalyst (Scheme 8c) [58]. [Et_4_N]_3_[CYA] was the main component of the most effective catalyst among these organocatalysts. Notably, this was the first reported example of direct organocatalytic conversion of low-concentration CO_2_ in air (0.04% *v*/*v*) for 2-oxazolidinone synthesis. It could be ascribed to the better activation of CO_2_ in the presence of [H_3-n_CYA]^n−^ (n = 1−3) than that of [Phth]^−^ and [2-PyO]^−^ anions (Scheme 8e). Besides, triethanolamine (TEOA) as a low-cost and biodegradable alkanolamine showed excellent performance on affording 2-oxazolidinones (Scheme 8d) [59]. As can be seen from Scheme 8f, TEOA activated CO_2_ to form a ring-shaped carbonate intermediate, which could be beneficial to the reaction procedure.

It is worth mentioning that allylamines are less active than propargyl amines in the reaction with CO_2_ because C≡C bonds are more prone to being attacked by carbamates than C=C bonds. However, the carboxylative cyclization of allylamines and CO_2_ would significantly expand the toolbox of 2-oxazolidinones synthesis. Most recently, Yu and co-workers summarized recent progress on this reaction by homogeneous catalysis in detail [60]. In addition, they also reviewed various cyclization reactions of allenic amines with CO_2_.

#### 2.2.2. Direct/Indirect Condensation of Amino Alcohols and Carbon Dioxide

In 2013, there were two excellent reviews dealing with the synthesis of 2-oxazolidinones through carbonylation of corresponding amino alcohols with CO_2_ (before 2013) by Pulla’s [14] and Tomishige’s [15] groups. Recently, Fekri et al. provided a review of recent developments (before 2017) in the synthesis of 2-oxazolidinone derivatives via dehydrative condensation of β-amino alcohols and CO_2_ with a discussion on the mechanistic aspects of the reactions in detail [61]. Based on the above works, this review will briefly discuss only the most recent literature (2017–2018) on direct/indirect condensation of amino alcohols and CO_2_.

**Scheme 9:**
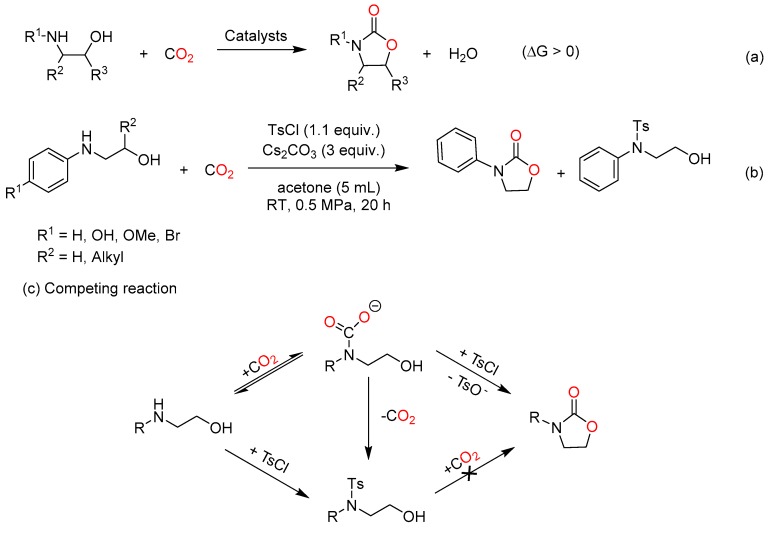
Synthesis of 2-oxazolidinones through carbonylation of amino alcohols with CO_2._

Despite efficient processes, the direct condensation of amino alcohols and CO_2_ were limited by harsh conditions (high temperature and pressure) and side reactions [62,63,64]. This could be attributed to the thermodynamic limitation of the reaction with water as by-product (Scheme 9a). Hence, organic bases, electrophiles and CeO_2_ were used for removing water from the system in most cases to improve the yield of products [65,66].

In 2018, Repo et al. developed an efficient one-pot approach using an external base and *p*-toluenesulfonyl chloride (TsCl) as a hydroxyl group activating reagent to synthesize cyclic carbamates from CO_2_ and amino alcohols (Scheme 9b) [67]. This stoichiometric reaction system allowed 2-oxazolidinones with the 3-aryl-5-alkyl substitution pattern to be obtained under mild conditions in good yields and high enantiomeric excess. In the reaction (Scheme 9c), a carbamate salt is formed from CO_2_ and amino alcohol, followed by the tosylation of the -OH to improve its leaving group character. Finally, ideal product is obtained via a ring-closing process. However, the carbamate species must be formed firstly and stabilized to avoid the competition of *N*-tosylation reaction. Unlike the above base-promoted work, a series of K-La-MgO with different K loadings (1, 3, 5, and 7 wt%) synthesized by combustion were screened for carbonylation of diethanolamine in a green and clean route [68]. Among them, 5% K-La-MgO was found to show the best performance. The catalytic process with 5% K-La-MgO offered 72% conversion of diethanol amine and 100% selectivity of 3-(2-hydroxyethyl)-1,3-oxazolidin-2-one at 150 °C and 2.0 MPa CO_2_ pressure.

**Scheme 10:**
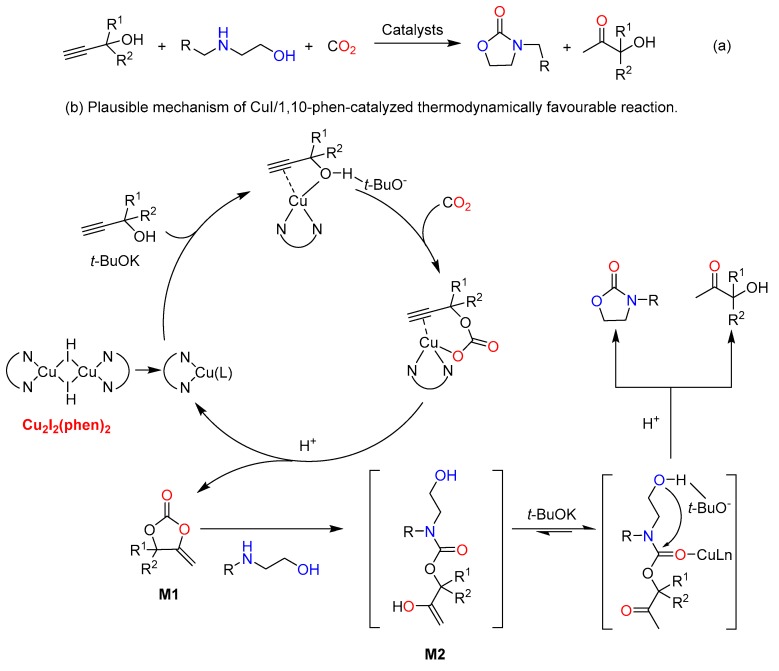
Synthesis of 2-oxazolidinones through thermodynamically favourable approach [68].

Additionally, a thermodynamically favourable one-pot three-component approach of propargylic alcohols, CO_2_ and 2-aminoethanols was described by He et al. in 2016 at first, offering 2-oxazolidinones with equal amount of α-hydroxyl ketone (Scheme 10a). As shown in Table 2, organic solvent, high CO_2_ pressure and excess propargylic alcohols were indispensable in the Ag-based catalytic system to achieve excellent yields [69,70], while in the CuI/1,10-phen-catalyzed three-component reaction, the desired products in good to excellent yields were obtained under mild reaction conditions without solvent. It was worth mentioning that cheap, commercially available copper catalysts, and the ability of C≡C activation have been widely utilized in CO_2_ transformations. The reaction consists of two steps. Firstly, α-alkylidene cyclic carbonate intermediate **M1** is formed via the carboxylative cyclization of propargylic alcohol and CO_2_. Then, **M1** undertakes a nucleophilic addition by a 2-aminoalcohol to form the corresponding β-oxopropylcarbamate species **M2**, which simultaneously affords the 2-oxazolidinone and an α-hydroxyketone with a subsequent intramolecular cyclization (Scheme 10b). Notably, copper complex Cu_2_I_2_(phen)_2_ in situ formed from CuI and 1,10-phen could activate the C≡C through coordination. Most recently, 1,5,7-triazabicylo[4.4.0]dec-5-ene ([TBDH][TFE]) trifluoroethanol was used to catalyze the synthesis of oxazolidinones from propargylic alcohols, 2-aminoethanols and CO_2_ under atmospheric pressure [71]. The catalysts could be recovered and reused at least five times without obvious loss of activity.

### 2.3. Quinazoline-2,4(1H,3H)-Diones through Carbonylation of 2-aminobenzonitrile and CO_2_

Quinazoline-2,4(1*H*,3*H*)-diones have wide applications in the synthesis of pharmaceuticals, such as Zenarestat, Prazosin, Doxazosin, and Bunazosin [72]. The synthesis of quinazoline-2,4(1*H,*3*H*)-dione derivatives through carbonylation of 2-aminobenzonitrile and CO_2_ is an environmentally benign process with 100% atom efficiency. This reaction was highlighted by Soleimani-Amiri et al. [72]. They classified the reactions based on the type of catalysts to review the works before 2017. Thus, we continued to summarise the published work in recent two years.

**Scheme 11:**
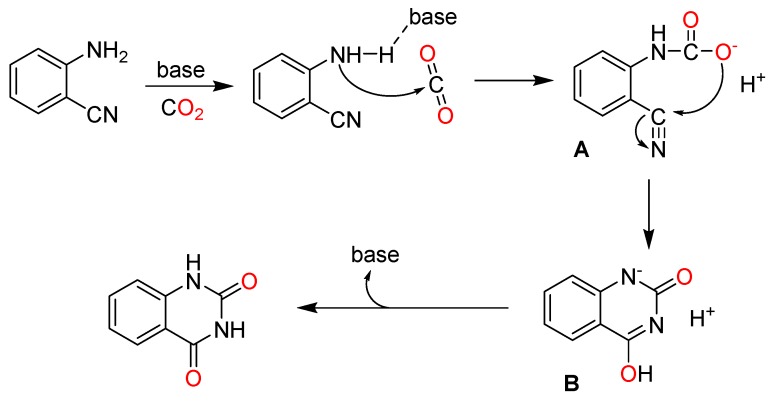
Possible reaction pathway of 2-aminobenzonitrile and CO_2._

According to the previous literature [73,74,75], amino group of 2-aminobenzonitrile is initially activated by basic catalysts, which facilitates the nucleophilic attack of 2-aminobenzonitrile at CO_2_ to generate intermediate **A**. Then, nucleophilic cyclization of **A** produces **B**. Finally, **B** converts into quinazoline-2,4(1*H,*3*H*)-dione with the regeneration of the catalysts (Scheme 11).

**Scheme 12:**
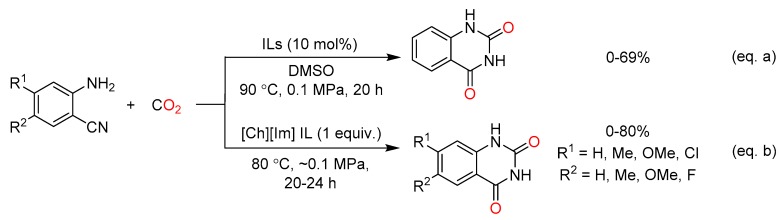
IL-catalyzed transformation of 2-aminobenzonitrile and CO_2._

Undeniably, numerous IL-based systems [73,74,76,77,78] exhibit excellent performance in the transformation of 2-aminobenzonitrile and CO_2_ [79]. However, almost all the reported IL systems involve high usage amounts of dual catalyst-solvent media (>1.0 equiv.). There were no intensive and systematic research figuring out the role of the IL, such as the effects of cation and anion until Dyson’s work [80] and Wang’s work [81] was published (Scheme 12).

Dyson et al. found that the cation from the IL did not play a direct role in the activation of 2-aminobenzonitrile, CO_2_, or the intermediates in the catalytic cycle [80]. On the contrary, the basicity of the anion had an inseparable relationship with the reaction rate. Notably, the acidity of quinazoline-2,4(1*H,*3*H*)-dione resulted in its deprotonation in basic catalytic conditions with the formation of quinazolide anion. Furthermore, the reaction was limited due to the neutralization of the basic IL catalyst and quinazolide anion.

**Figure 4:**
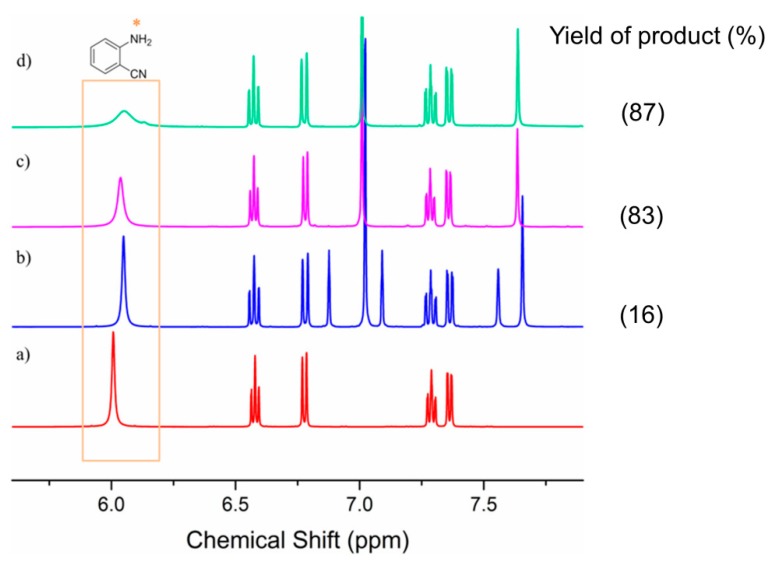
^1^H-NMR spectra of (**a**) 2-aminobenzonitrile with (**b**) [HMIm][Im] (pKa value of cation was 7.1), (**c**) [HDBU][Im] (pKa value of cation was 11.7) or (**d**) [HMTBD][Im] (pKa value of cation was 13.0). The H signal of amino in 2-aminobenzonitrile became broader when the basicity of cation was higher. Reprinted with permission from [81] (Copyright 2018 American Chemical Society).

**Scheme 13:**
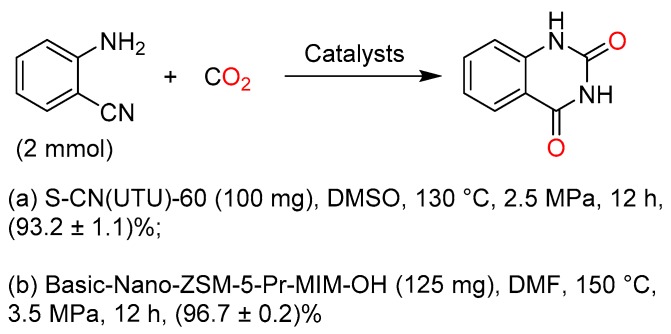
Synthesis of quinazoline-2,4(1*H,*3*H*)-dione by heterogeneous catalysts.

On the other hand, according to quantum-chemical calculations, NMR spectroscopic investigations and controlled experiments, Wang et al. proposed that the basicity of cation affects the catalytic activity of ILs dramatically (Figure 4) [81]. In addition, the cation with more OH groups exhibited poorer catalytic activity. Therefore, a hydroxyl functionalized aprotic IL [Ch][Im] was designed and exhibited high catalytic activity (Scheme 13b).

Inspired by Dyson’s work, Fujita et al. found that *^n^*Bu_4_NF was an effective quaternary ammonium salt for catalyzing the cyclization of 2-aminobenzonitriles when a catalyst loading of 1 mol% was used at 110 °C under CO_2_ pressure of 2 MPa for 24 h [56].

As introduced in Section 2.2.1 of this review, cyanuric acid-based organocatalystw could be good activators for CO_2_ capture and utilization [58]. [Et_4_N]_3_[CYA] was also an efficient organocatalyst in converting atmospheric pressure of CO_2_ into quinazoline-2,4(1*H*,3*H*)-diones without any external base or promoter.

In recent years, great efforts have been made to develop heterogeneous catalysts for a greener procedure to quinazoline-2,4(1*H,*3*H*)-dione. Both a graphitic carbon nitride catalyst [82] and a functionalized basic nanocrystalline zeolite [83] prepared by Srivastava et al. were demonstrated to be effective in the synthesis of quinazoline-2,4(1*H,*3*H*)-dione from the reaction of 2-aminobenzonitrile and CO_2_ (Scheme 13).

**Scheme 14:**
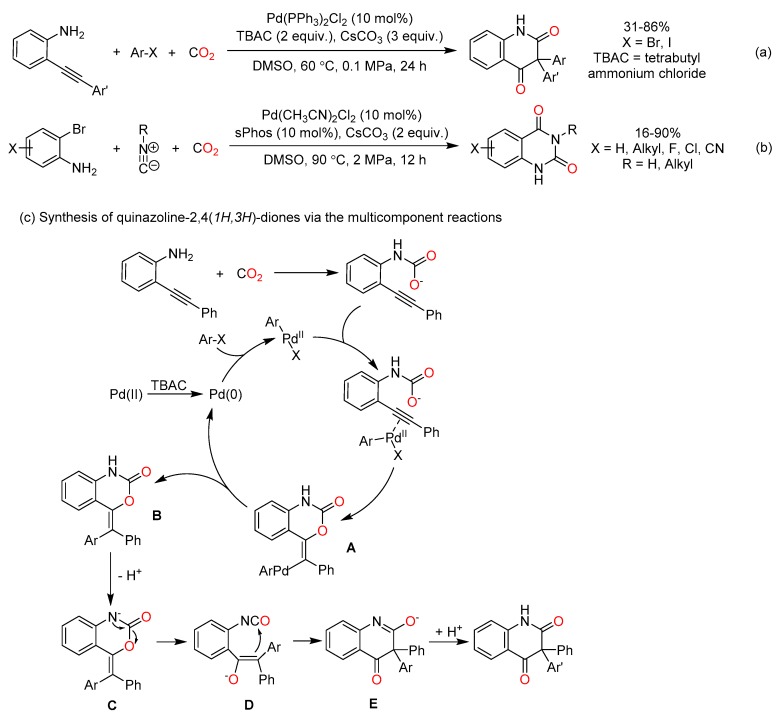
Multicomponent reactions including CO_2_ to synthesize quinazoline-2,4(1*H*,3*H*)-diones.

In recent years, much attention was paid to the synthesis of quinazoline-2,4(1*H*,3*H*)-diones via the multicomponent reactions involving CO_2_ (Scheme 14). In 2017, Cheng et al. reported palladium-catalyzed multicomponent reactions of *O*-alkynylanlines, aryl iodides, and atmospheric pressure of CO_2_, generating 3,3-diaryl 2,4-quinolinediones [84]. This reaction provided a one pot strategy to incorporate CO_2_ into heterocycles bearing a quaternary carbon center (Scheme 14a). As shown in Scheme 14c, the carboxylation of amine with CO_2_ and the *trans*-oxopalladation of C≡C bond by ArPd^II^X species sequential occur to afford intermediate **A**. Subsequently, intermediate **A** undergoes an reductive elimination, deprotonation and rearrangements to generate the product. Moreover, palladium-catalyzed cyclization reactions of *O*-haloanilines, CO_2_ and isocyanides were employed to prepare *N*3-substituted quinazoline-2,4(1*H,*3*H*)-dione with moderate to excellent yields (Scheme 14b) [85].

### 2.4. Urea Derivatives from Amine Derivatives and CO_2_

It is well known that urea derivatives have a wide range of applications in pharmaceutical chemistry, organic synthesis as well as analytical chemistry [86,87]. Traditionally, urea derivatives were synthesized by the stoichiometric reaction of amines and isocyanates [88]. Moreover, the reaction of amines and highly toxic phosgene/phosgene analogs was performed to afford urea derivatives [89]. Besides, catalytic oxidative carbonylation of amines in the presence of CO was also developed to obtain ureas [90]. Notably, alternative synthetic routes of CO_2_ as a source with amines have attracted considerable attention in recent years.

**Scheme 15:**
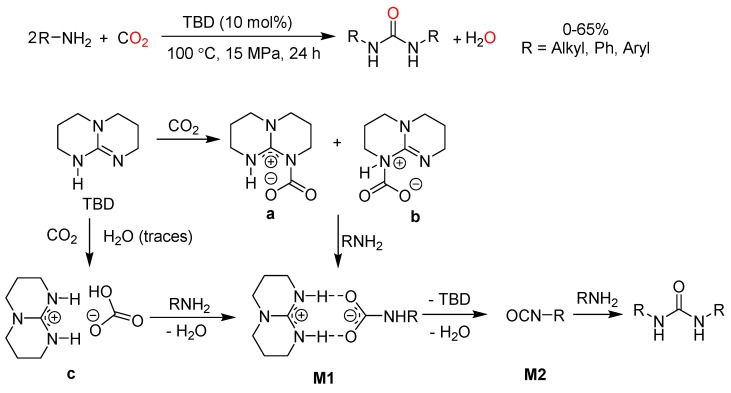
Bicyclic guanidine-catalyzed urea formation.

In the last decades, bicyclic guanidines, as important organic bases, have shown to be active catalysts in chemical fixation of CO_2_. For example, *N*,*N′*-dialkylureas were synthesized via the carbonylation of amines with CO_2_ using TBD as catalyst (Scheme 15) [91]. Among three TBD-CO_2_ species (**a**–**c**) shown in Scheme 15, the formation of bicarbonate-guanidinium salt **c** is ascribed to trace of water. The reaction proceeds with a sequential electrophilic attack of amine on **c**, elimination of water from **M1** and reaction between **M2** and amine leading to *N,N′*-dialkylureas.

**Figure 5:**
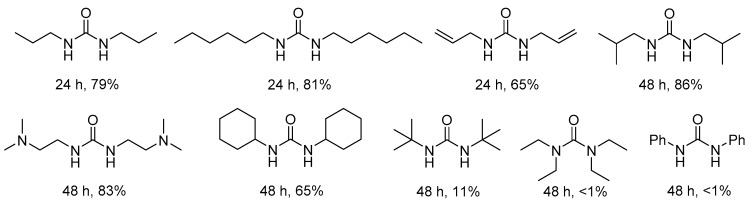
Various ureas by CeO_2_/NMP system. Reprinted with permission from [92] (Copyright 2015 Elsevier Inc.).

The use of metal oxides and metal salts of oxalates significantly paved the way for *N,N’*-dialkylurea synthesis in recent years (Table 3). CeO_2_ showed high activity for the direct synthesis of 1,3-dibutylurea from CO_2_ and *n*-butylamine [92]. The catalytic system was applicable to various amines such as linear primary alkylamines and branched primary alkylamines (Figure 5). Meanwhile, secondary amines and aniline, which were unreactive using CeO_2_ in *N*-methyl-2-pirrolidinone (NMP), were also transformed into the corresponding ureas when 2-cyanopyridine was combined with CeO_2_ in NMP. 

Y_2_(C_2_O_4_)_3_ and Y_0.08_Zr_0.92_O_1.96_ mixed oxide were independently used to catalyze carbonylation of aliphatic primary amines with CO_2_. Y_2_(C_2_O_4_)_3_ provided the highest yield of *N*,*N′*-dialkylurea among various metal salts of oxalates (Zr(C_2_O_4_)_2_, Ce_2_(C_2_O_4_)_3_, Mn(C_2_O_4_), Na_2_(C_2_O_4_), Ni(C_2_O_4_)) [93]. Y_0.08_Zr_0.92_O_1.96_ mixed oxide as the heterogeneous catalyst showed higher catalytic activity than Y_2_(C_2_O_4_)_3_ [94]. The presence of oxygen vacancies in the mixed oxide was of great importance for adsorption and activation of CO_2_ in the carbonylation process. It should be ascribed to the additional reduction potential derived from the oxygen vacancies for the reduction of CO_2_ to CO and/or surface carbonaceous species (an important intermediate).

Indium-catalyzed transformations of CO_2_ with aliphatic and aromatic silylamines have been exploited by Stephan et al. [95]. The indium compound In(N(SiMe_3_)_2_)Cl_2_·(THF)_n_ (THF = tetrahydrofuran) was able to efficiently catalyze the reaction and afford a wide range of aryl and alkyl ureas with 0.05–5 mol% catalyst loadings. Notably, the available main group metal-catalyzed transformations of CO_2_ were improved.

**Scheme 16:**
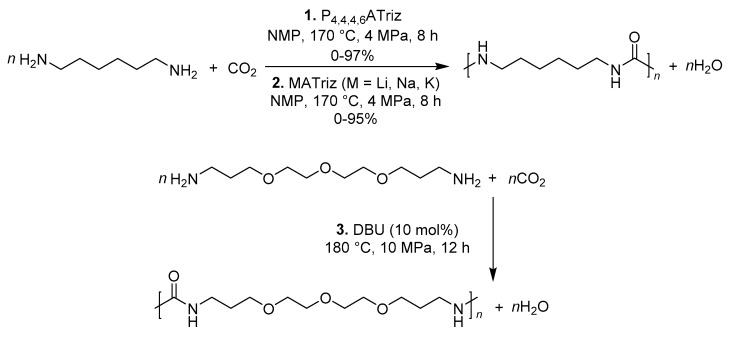
Synthesis of polyureas via the polymerization of CO_2_ with diamines.

Polyureas containing urea linkages and connected by hydrogen bonds are a new kind of polymer. In 2016, good to excellent yields of various polyureas were achieved with different diamines and CO_2_ by hexyltributylphosphonium aminotriazole (P_4,4,4,6_ATriz, ATriz = 3-amino-1*H*-1,2,4-triazole) (**1**, Scheme 16) IL catalyst [96]. It was found that the catalytic performance was essentially consistent with the basicity of ILs described by Deng et al. Recently, some functional polyureas were successfully synthesized via the polymerization of CO_2_ with diamines using amino triazole alkali salts i.e., MATriz as catalysts (**2**, Scheme 16), and M was Li, Na, K [97]. More importantly, the catalysts could be reused for several times without obvious deactivation.

**Figure 6:**
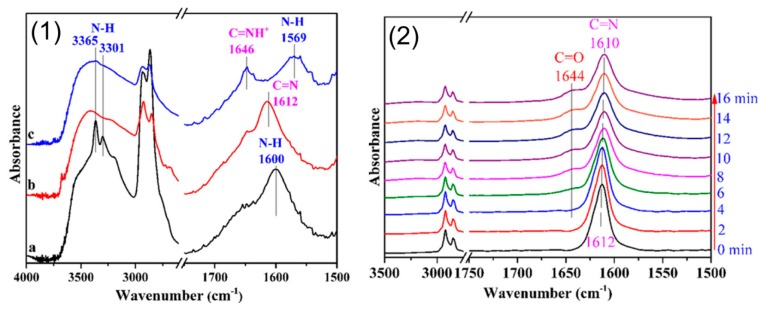
(**1**) FTIR spectra of (a) TOTDDA, (b) DBU, and (c) mixture of TOTDDA and DBU (TOTDDA:DBU was 10:1). The protonation of DBU by TOTDDA indicated that DBU could activate the -NH_2_ group of TOTDDA. (**2**) In situ ATR-FTIR spectra of DBU (C=N) and DBU·CO_2_ (C=O) collected at different periods of reaction time. The new band at 1644 cm^−1^ is assignable to C=O and this implies the formation of the complex of DBU·CO_2_. Reaction conditions: DBU 2 mmol, CO_2_ 8 MPa, 60 °C. Reprinted with permission from [98] (Copyright 2018 John Wiley and Sons).

Additionally, Arai et al. found that DBU (**3**, Scheme 16) was the most active among multiple organic and inorganic base catalysts in addition of CO_2_ to 4,7,10-trioxa-1,13-tridecanediamine (TOTDDA) [98]. DBU could activate both CO_2_ and -NH_2_ group of TOTDDA, which was demonstrated by in situ high-pressure attenuated total reflectance Fourier transform infrared spectroscopy (ATR-FTIR), resulting in a high catalytic performance (Figure 6).

### 2.5. Novel Synthetic Strategies of Carbamate Derivatives Based on Amines and CO_2_

#### 2.5.1. *N*-Tosylhydrazones as a Building Block to Construct C-N Bond

**Scheme 17:**
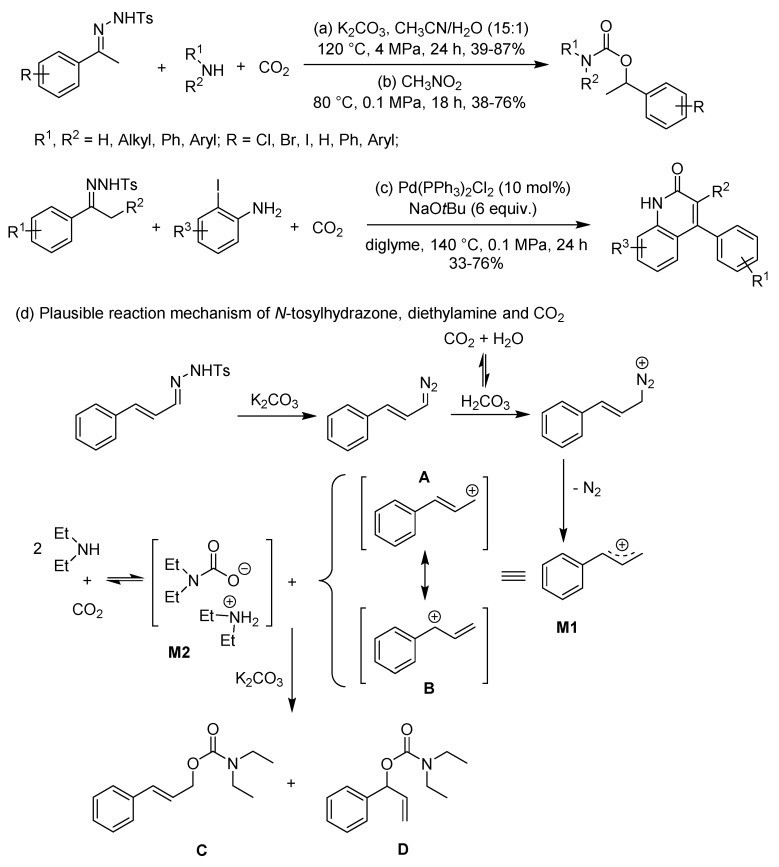
*N*-tosylhydrazones as a building block to construct C-N bonds.

*N*-Tosylhydrazones have been extensively employed as useful building blocks to construct complicated molecules. In 2015, Jiang et al. reported the first approach for the synthesis of organic carbamates by a three-component coupling reaction of CO_2_, amines, and *N*-tosylhydrazones (Scheme 17a) [99]. Notably, in the presence of K_2_CO_3_ and a mixed CH_3_CN/H_2_O solvent, the reaction system tolerates a variety of valuable functional groups on the products, which provided ample potential for further organic syntheses. Subsequently, Chung et al. modified the procedure performed under mild conditions with desired carbamates (Scheme 17b) [100]. Nevertheless, poor yields were observed compared with Jiang’s work. 

At present, however, there is insufficient evidence for the proposed mechanism [99]. Presumably, for example, *N*-tosylhydrazone decomposes in the presence of K_2_CO_3_, and generates diazo compound, which can be followed by a protonation and a thermodynamically favorable liberation of dinitrogen to give allylic carbocation species **M1** (a resonance hybrid of **A** and **B**) (Scheme 17d). Finally, the carbamate anion of **M2** as a nucleophile attacks to **M1** generating products of **C** and **D**.

Notably, Cheng et al. developed a strategy towards 4-aryl-2-quinolinones by a three-component coupling reaction of *N*-tosylhydrazones, 2-iodoanilines and CO_2_ catalyzed by Pd^II^/phosphine system, which was benefited from the *N*-tosylhydrazone chemistry (Scheme 17c) [101]. Gratifyingly, four novel bonds: two C-C, one C=C and one C-N formed simultaneously in the heterocycles.

#### 2.5.2. *O*-Aryl Carbamates from Three-Component Reactions Containing Amines and CO_2_

**Scheme 18:**
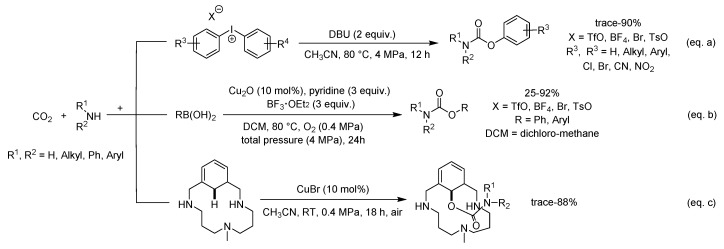
Synthesis of *O*-aryl carbamates by three-component reactions containing amines and CO_2._

A straightforward route to *O*-aryl carbamates was achieved through a three-component reaction of CO_2_, amines and diaryliodonium salts with an organic base (DBU) as the promoter (Scheme 18a) [102]. In this phosgene- and metal-free protocol, diaryliodonium salts was used as electrophilic arylating agents to trap the in situ-generated carbamate anions. However, this transformation generated a stoichiometric amount of by-product, i.e., aryl iodides. In addition, the preformation of diaryliodonium salts was required. 

Additionally, a more convenient route to *O*-aryl carbamates by a copper(I)-catalyzed oxidative coupling reaction between arylboronic acids, amines and CO_2_ was developed (Scheme 18b) [103]. At the same time, BF_3_·OEt_2_ was employed as the promoter and O_2_ as the oxidant. This approach tolerated a wide range of functionalized *O*-aryl carbamates.

Ribas and Company et al. reported a one-pot procedure of a model pincer-like arene, amines and CO_2_ to synthesize *O*-arylcarbamates (Scheme 18c) [104]. This reaction performed smoothly at room temperature by an aerobic Cu(II)-based catalyst and a nucleophile of carbamic ammonium salts in situ generated from amines and CO_2_ gas.

#### 2.5.3. *N*-Arylcarbamate from Three-Component Reactions Containing Amines and CO_2_

**Scheme 19:**
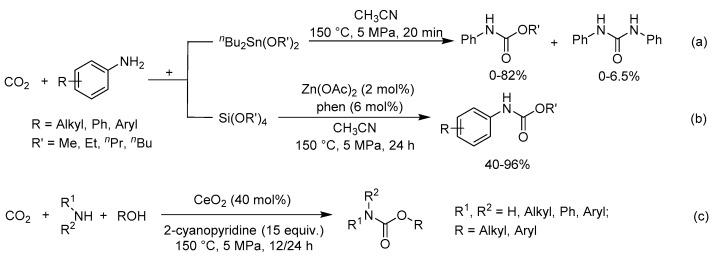
Syntheses of *N*-arylcarbamates via various strategies.

**Figure 7:**
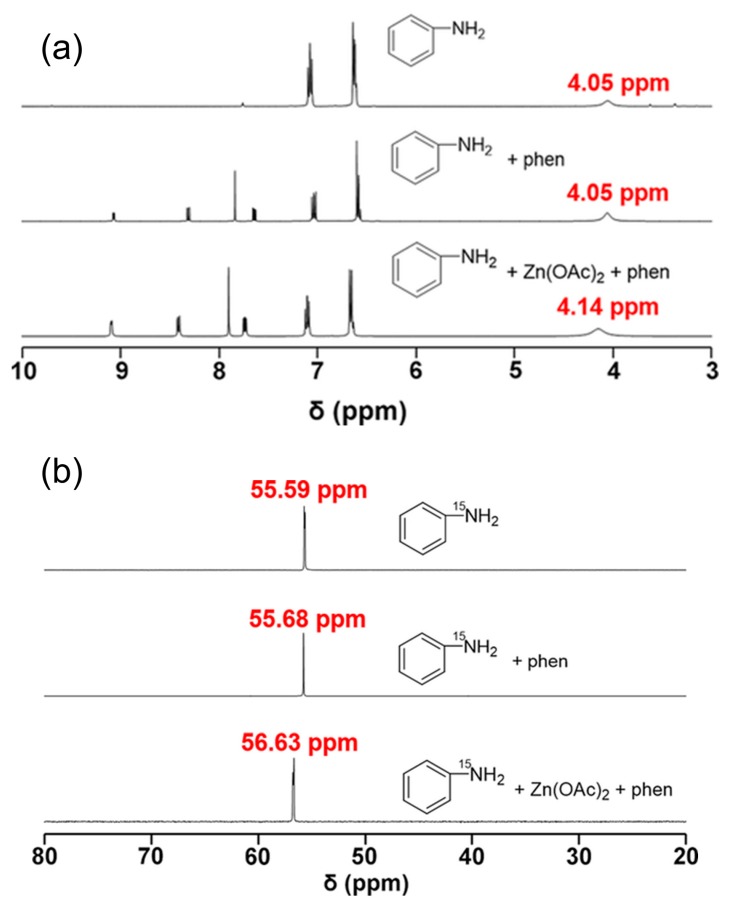
(a) ^1^H- and (b) ^15^N-NMR spectra of aniline with and without Zn(OAc)_2_/phen in CD_3_CN at room temperature. The chemical shifts in both ^1^H and ^15^N-NMR spectra illustrated the interaction between aniline and acetate, and the formation of hydrogen bonds. Reprinted with permission from ref. [107] (Copyright 2017 John Wiley and Sons).

*N*-Arylcarbamate synthesis directly from CO_2_, amines and organic halides often has negative environmental effects [105]. Choi et al. reported the first successful example of a halogen-free process for producing *N*-phenylcarbamate from CO_2_, aniline and dibutyltin dialkoxide (Scheme 19a) [106]. Gratifyingly, the corresponding methyl *N*-phenylcarbamate was formed in a yield of 80% along with *N′*-diphenylurea as by-product. Recently, they found that silicate ester (i.e., Si(OR)_4_) could be incorporated with aliphatic or aromatic amines and CO_2_ to synthesize *N*-arylcarbamate (Scheme 19b) [107]. Notably, the corresponding carbamates of aromatic amines could be obtained in a yield of up to 96%. Besides, the reaction was chemoselective toward amine activation. The use of Zn(OAc)_2_/1,10-phenanthroline (phen) catalysts offered the best performance because of the carboxylate-assisted proton activation, as shown in Figure 7.

Alternatively, adding the corresponding alcohols to the system including CO_2_ and amines was also an efficient route to obtain *N*-arylcarbamates. In 2018, the combination of CeO_2_ and 2-cyanopyridine was used in the direct synthesis of *N*-arylcarbamates from CO_2_, amines and alcohols (Scheme 19c) [108]. Various linear and branched alcohols were transformed into the corresponding carbamates in high selectivities.

#### 2.5.4. Allyl Carbamates from Amines and CO_2_

**Scheme 20:**
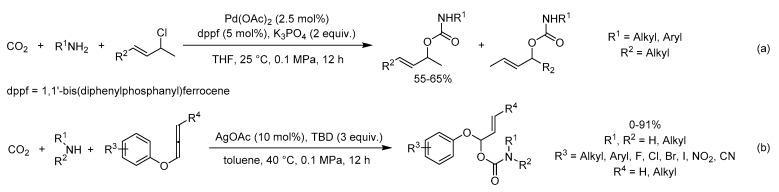
Synthesis of allyl carbamates from amines and CO_2._

As seen from Scheme 20a, Zhao et al. developed a three-component reaction of unsymmetrical allylic chlorides, CO_2_ and amines to afford allyl carbamates by Pd catalysis [109]. This method tolerated various allyl carbamates in moderate yields but with excellent regioselectivity (99/1).

A facile and efficient methodology for the synthesis of new acetal-type *O*-allyl carbamates by regioselective coupling reaction of CO_2_, amines and aryloxyallenes was explored in 2018 (Scheme 20b) [110]. A wide range of desired products were obtained in moderate to excellent yields using the catalytic system of AgOAc and TBD. Jiang et al. found that a cationic species generated from Ag(I) salt and TBD was the key active complex in the transformation, which was indicated by ^1^H-NMR studies.

## 3. C-N Bond Formation through CO_2_ Hydrogenation

For the past fifteen years, reductive methylation using N-site-based nucleophiles and CO_2_ to give the corresponding methyl amines or formamide, which represents one of the most economical and environmentally friendly routes for C-N bond formation using CO_2_ as C_1_ building block has been investigated intensively. Recently, in this hot field, several groups such as Cantat (2015, reductive functionalization of CO_2_ with amines) [111,112], Yan (2015, transition metal-catalyzed methylation) [113], Beller (2017, catalytic methylation) [114], He (2018, transition-metal-free catalysis) [115], Kühn (2018, an update of CO_2_ catalytic conversion) [116], Fernández-Alvarez and Oro (2018, homogeneous catalytic reduction of CO_2_ with silicon-hydrides) [117], Motokura (2018, organocatalysis with silanes) [118], have reviewed the recent developments in CO_2_ hydrogenation based on amine substrates from different viewpoints. On the basis of the previous reviews and the integrity of this article, here, the introduction of the development of CO_2_ conversion based on amines will be emphasized in a special view. The contents of this part include the regulatory strategies for functionalization of CO_2_ for N-methylation and N-formylation of amines with phenylsilane and heterogeneous catalysis N-methylation of amines with CO_2_ and H_2_.

### 3.1. Regulatory Functionalization of CO_2_ with Amines and Phenylsilane: N-Methylation and N-Formylation of Amines

**Scheme 21:**
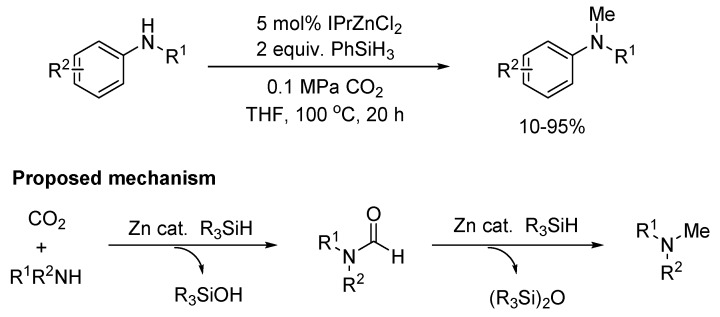
Zinc complex-catalyzed methylation of amines using hydrosilanes.

In 2013, the methylation of amines was achieved using hydrosilanes as reductants through zinc complex catalysis by the Cantat group (Scheme 21) [119]. A mechanism going through a formamide intermediate was proposed. In recent years, many researchers undertook the study on selective catalysis of *N*-methylation and *N*-formylation of amines from amines, CO_2_, and hydrosilane.

**Scheme 22:**
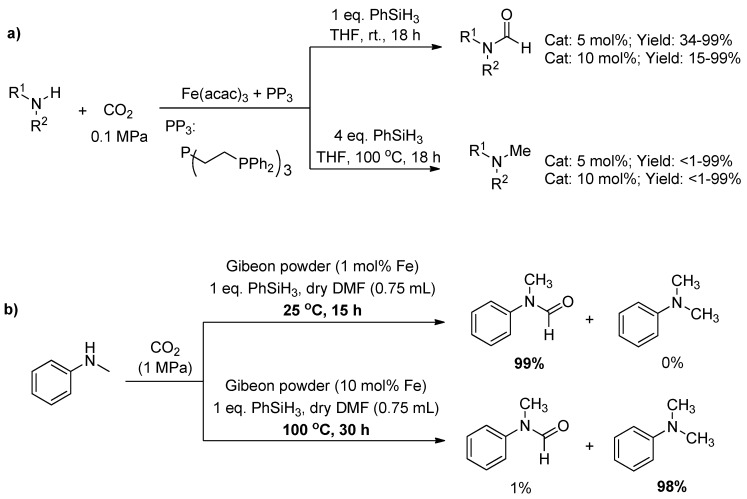
Fe-based catalysis for temperature controlled *N*-formylation/*N*-methylation.

Subsequently, they described the first iron catalysts able to promote the reductive functionalization of CO_2_ with amines using hydrosilanes as reductants to formamide and methylamine derivatives under mild reaction conditions (Scheme 22a) [120]. At room temperature, the chemoselectivity of formamide was almost 100%. After increasing the temperature to 100 °C, the selectivity of *N*-methylation was greatly improved and the majority methylamine derivatives were obtained under the elevated loading of catalyst. Similarly, iron-rich natural mineral Gibeon meteorite was used as an efficient heterogeneous catalyst for the *N*-formylation or *N*-methylation of amines with CO_2_ and hydrosilanes recently (Scheme 22b) [121]. In the work, a wide range of amines was converted into their corresponding formamides in high yields at room temperature. By simply varying the conditions e.g., increasing the temperature (25→100 °C) and catalytic loading (1→10 mol%), the selectivity was switched from *N*-formylated to *N*-methylated products. Moreover, the Gibeon meteorite catalyst was recycled and reused at least five times without appreciable activity loss. 

**Scheme 23:**
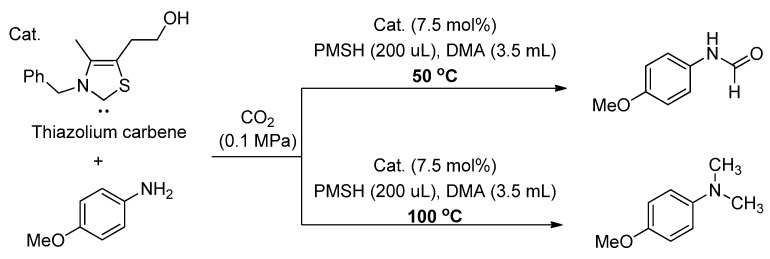
Thiazolium carbene-based catalyst for *N*-formylation/*N*-methylation.

In 2015, the Dyson group reported an effective thiazolium carbine-based catalyst for the N-formylation of amines, using polymethylhydrosiloxane (PMHS) as a reducing reagent under ambient pressure [122]. A board range of primary amines could be converted into the corresponding formamides with PMHS (200–300 mL) at 50 °C under atmospheric pressure of CO_2_. Notably, a interesting discovery, namely that the variation of reaction temperature could change the reaction products, was also made. For example, the *N*-formylation could be turned into *N*-methylation by changing the reaction temperature from 50 to 100 °C with PMHS (200–300 mL), and methylamine was obtained in high yield (Scheme 23).

**Scheme 24:**
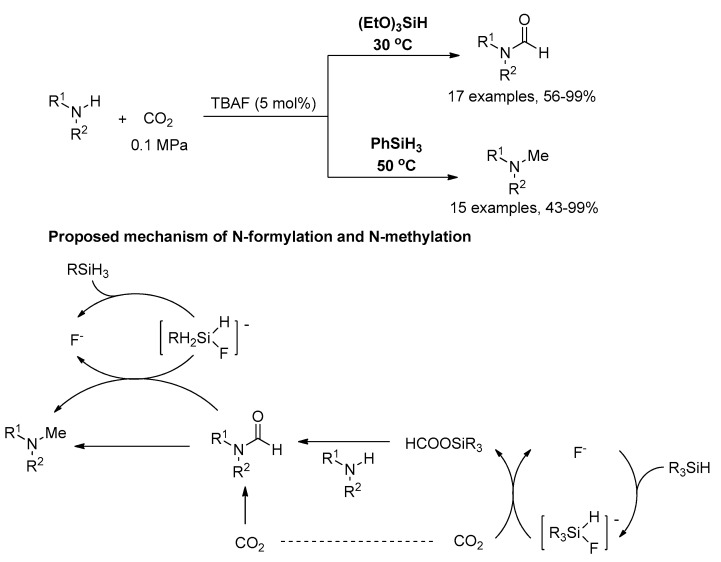
TBAF catalysis for selective *N*-formylation/*N*-methylation.

Subsequently, the He group [123], Fu group and Lin group [124] reported the selective synthesis of formamides or methylamines from amines, CO_2_, and hydrosilane under the different catalytic conditions in the same year. In He’s work, butylammonium fluoride (TBAF) was used for the reductive functionalization of CO_2_ with amines to selectively afford formamides or methylamines by employing different hydrosilanes (Scheme 24). Formamides were obtained with triethoxysilane as reductant, and methylamines with phenylsilane in excellent yield under atmospheric pressure of CO_2_ at 30 and 50 °C, respectively. The mechanism of formation of the key silyl formate intermediate in the formylation step and fluoride-promoted hydride transfer from the hydrosilane to CO_2_/formamide was proposed.

**Scheme 25:**
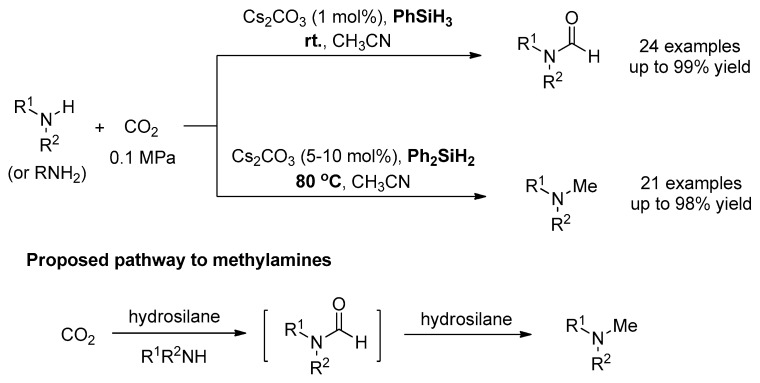
Cs_2_CO_3_ catalysis for selective *N*-formylation/*N*-methylation and proposed mechanism.

The Fu group and Lin group developed a simple and effective alkali-metal carbonate, i.e., cesium carbonate, that catalyzed both the formylation and methylation reactions under mild conditions (Scheme 25). Through varying the reaction temperature and loading of silane, the formylation/methylation selectivity could be conveniently controlled. By means of experimental and computational studies, they revealed the possible mechanism including: (i) activation of Si−H by Cs_2_CO_3_, (ii) insertion of CO_2_ into Si−H, (iii) formylation of amines by silyl formate, and (iv) reduction of formamides to methylamines. 

**Scheme 26:**
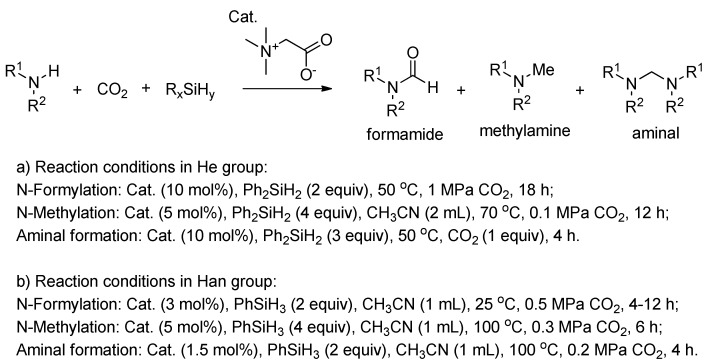
Glycine betaine-catalyzed selective *N*-formylation/*N*-methylation.

In 2007, the He group developed a glycine betaine-catalyzed strategy for the reaction of CO_2_ with amines and diphenylsilane (Scheme 26a) [125]. Through tuning the amount of CO_2_ and the reaction temperature, three kinds of products, i.e., formamide, methylamine, and aminal, were successively obtained. This scheme firstly achieved the hierarchical reduction of CO_2_ with amine and hydrosilane using organocatalysis with wide substrate scope. Almost at the same time, the Han group also reported the glycine betaine-catalyzed transformation of CO_2_ with amines to synthesize *N*-substituted compounds (Scheme 26b) [126]. Similarly, the selectivity to the reductive products could be controlled by the molar ratio of reactants (i.e., CO_2_, amines, and PhSiH_3_) and the temperature. Subsequently, they used lecithin as an organocatalyst for the formylation and methylation of various amines with CO_2_ to corresponding formamides and methylamines via using PhSiH_3_ as the hydrogen source [127]. Meanwhile, the product selectivity could also be easily controlled by the molar ratio of reactants and reaction temperature.

**Scheme 27:**
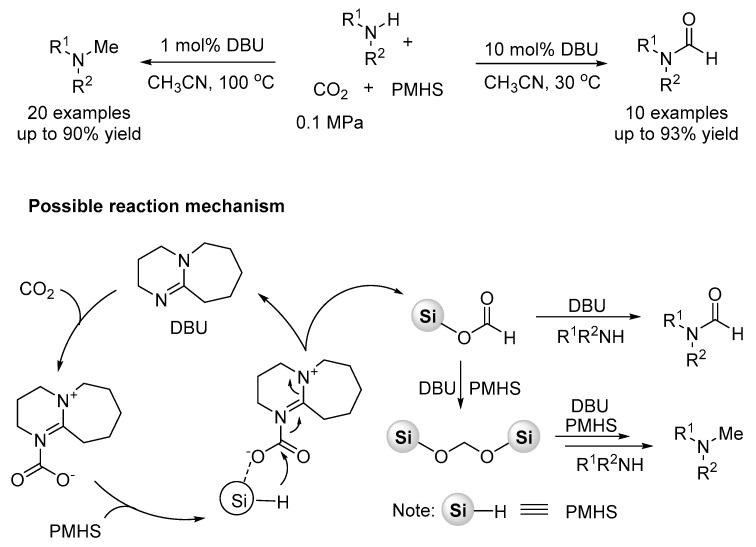
DBU-catalyzed selective *N*-methylation/N-formylation using PMHS as hydride reagent [128].

Recently, Chen and Xia reported an efficient DBU catalytic system for the selective *N*-methylation and *N*-formylation of amines with CO_2_ and PMHS (Scheme 27) [128]. The *N*-methylation products in high yields are obtained with 1 mol% DBU at 100 °C, and selective *N*-formylation of amines is realized with 10 mol% DBU at a lower temperature.

**Scheme 28:**
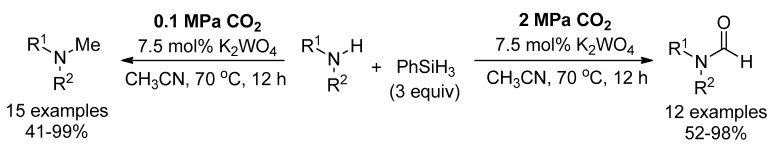
Pressure-switched *N*-methylation/*N*-formylation to methylamine/formamide.

In previous studes, the selective *N*-methylation and *N*-formylation of amines with CO_2_ was mainly dependent on the reaction temperature because higher reaction temperatures favour methylamine formation. Recently, a tungstate catalyst was employed for selective catalysis reductive functionalization of CO_2_ with amines and phenylsilane (Scheme 28) [129]. By varying the CO_2_ pressure, 2-electron or 6-electron reduction of CO_2_ was achieved to give formamides or methyl- amines, respectively. Meanwhile, the *N*-formylation proceeding through the silyl formate intermediate and the *N*-methylation through an aminal intermediate were demonstrated via control experiments and NMR studies.

**Scheme 29:**
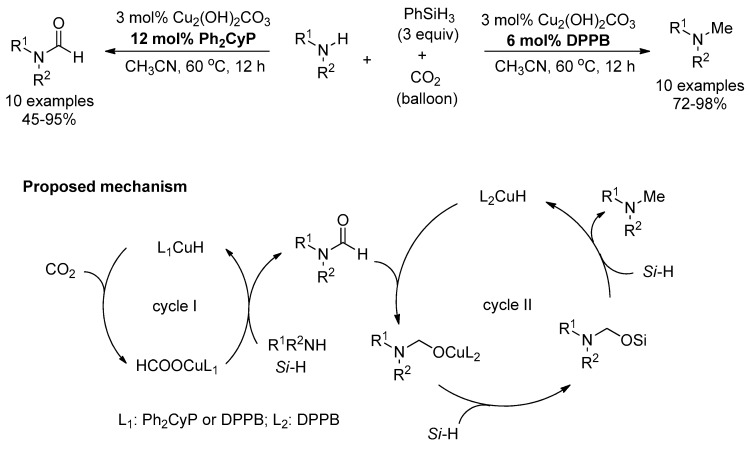
DBU-catalyzed selective *N*-methylation/*N*-formylation and possible mechanism [130].

Afterwards, Li and He studied the ligand-controlled copper complex-catalyzed selective transformations of amines and CO_2_ to formamides and methylamines with phenylsilane as reductant (Scheme 29) [130]. Using the same reaction conditions and copper compound, selective catalysis was accurately regulated through simply changing the phosphine ligand.

Initially, the coordination of copper with phosphine ligand Ph_2_CyP or DPPB led to the copper complex and the subsequent formation of the active Cu–H species, followed by CO_2_ insertion with the generation of the copper formate (Cycle I). Then, copper formate reacts with amine and hydrosilane to give the formamide along with the formation of the active Cu–H species (Cycle II). The catalytic ability of copper complex determines the final products through the catalytic cycle I or cycle II. The Cu(Ph_2_CyP) complex gave the final formamide product, and Cu(DPPB) could further catalyze the formamide reduction to the methylamine product.

#### The Mechanism of Metal and Organocatalysis

**Scheme 30:**
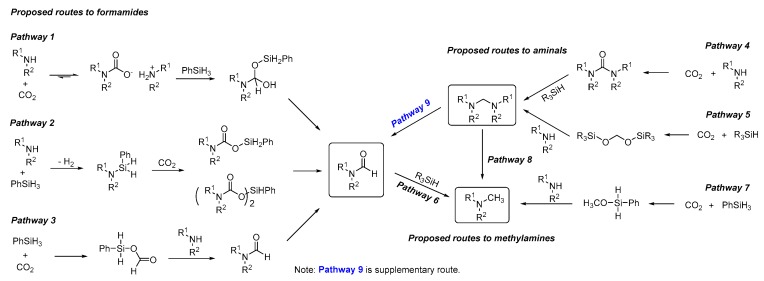
Proposed mechanistic pathways for *N*-methylation and *N*-formylation [131].

**Scheme 31:**
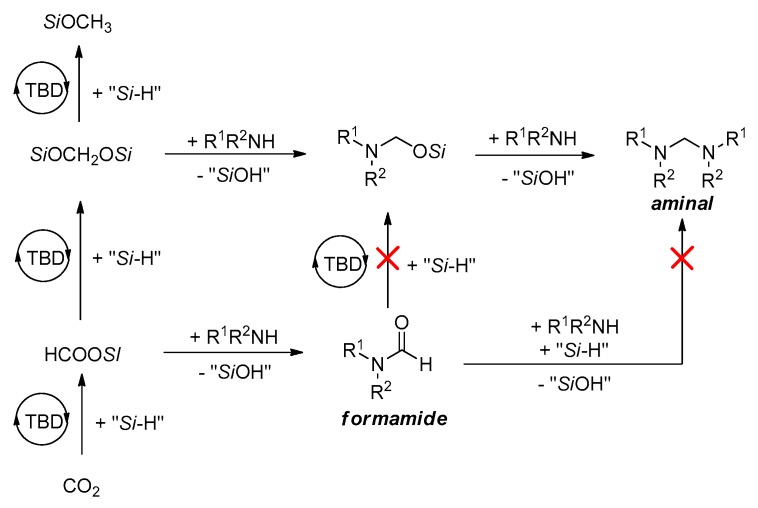
TBD-catalyzed aminal synthesis from amine, CO_2_ and hydrosilane [132].

**Scheme 32:**
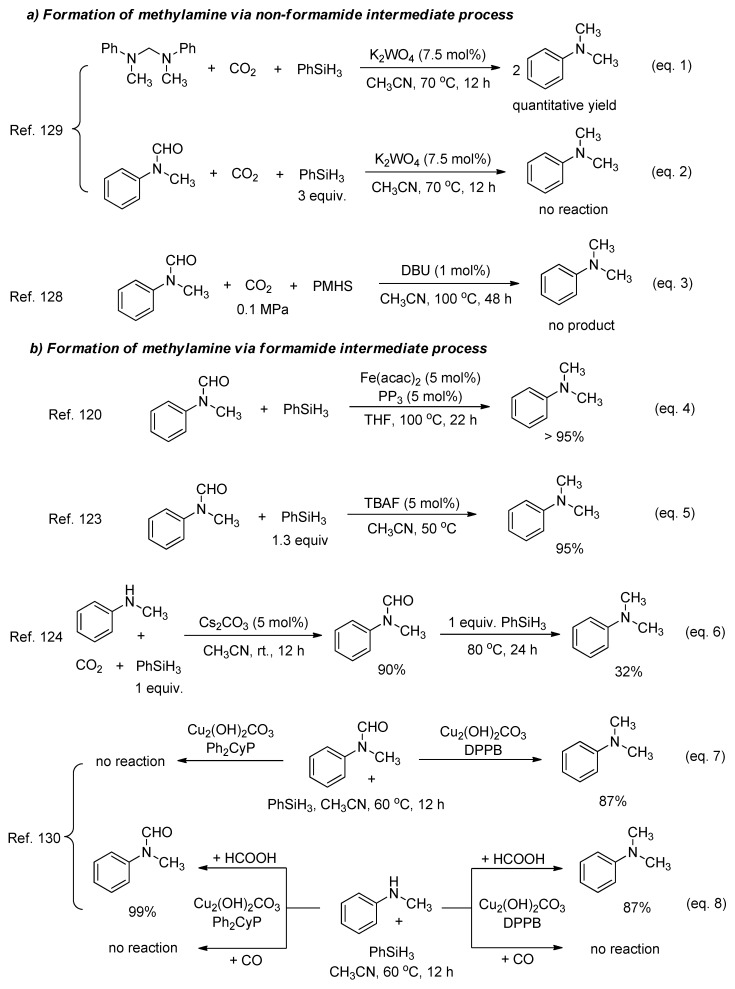
Summarized control experiments in the published work.

The previously proposed catalytic pathways for the reactions of *N*-methylation and *N*-formylation are summarized in Scheme 30 [131]. Among, the mechanisms for the synthesis of aminal from amine, CO_2_ and hydrosilane (Cantat’s TBD-catalyzed method) is similar to the methylation (Scheme 31) [132]. The aminal product is obtained via a [Si]OCH_2_O[Si] intermediate, and HC(=O)O[Si] intermediate does not react with amine to form formamide. Therefore, the formation of aminal does not undergo the formamide intermediate process. Besides, other organocatalysts including glycine betaine [125,126], DBU [128], K_2_WO_4_ [129], also supported the mechanism of aminal intermediate for the methylation of amines with CO_2_ using hydrosilane as reductant through detailed control experiments (Scheme 32, equations 1–3) and DFT study [133]. In addition, in some metal-based systems such as Fe [120], Cs [124], Cu [130], and a few organocatalysts (e.g., TBAF [123], lecithin [127], etc.), the experimental evidence supports the mechanism of further formamide hydrogenation to the corresponding the methylation under the corresponding conditions (Scheme 32, equations 4–8). In these works, control experiments, e.g., using the product formamide as reaction material, were carried out to demonstrate the pathway (Scheme 30).

**Scheme 33:**
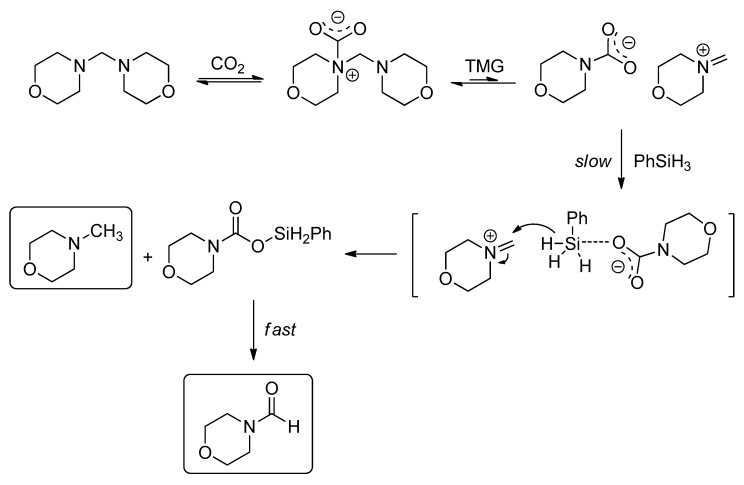
Proposed mechanism for reduction of aminal [131].

Recently, a mechanistic investigation was carried out by Nguyen group on the guanidine catalyzed reductive amination of CO_2_, using a combination of experimental and analysis method (^1^H, ^29^Si-NMR, FT-IR, MS, and GC profiling) (Scheme 33) [131]. The results showed that reduction of CO_2_ to formamide, aminal, and then N-methylamine is not sequential. Formamide was obtained dominantly at a lower temperature (23 °C), but an obvious increase of aminal, and then N-methylamine was detected at the higher temperature (60 °C).

Increasing the reaction temperature enables a competitive and higher-energy pathway to aminal and *N*-methylamine, which requires direct reduction of CO_2_ with PhSiH_3_ to formoxysilane PhSiH_2_OOCH intermediate. The theory provided a new evidence for the pathway to formamide from aminal (Scheme 33, pathway 9).

### 3.2. Heterogeneous Catalysis Methylation of Amines with CO_2_ and H_2_

The use of hydrosilane could lead to the formation of large amount of siloxanes as by-products which is not beneficial to the development of sustainable chemistry. Therefore, in place of hydrosilane with benign H_2_ would make the hydrogenation process cleaner. In addition, the advantages of heterogeneous catalyst such as easily recovery and recycle are obvious. Therefore, herein, heterogeneous catalysis on methylation of amines with CO_2_ and H_2_ was highlighted.

**Scheme 34:**
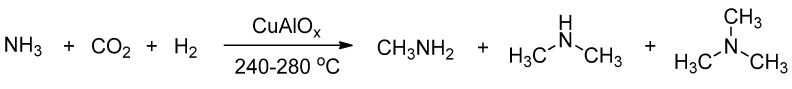
Heterogeneous metal-based catalysis methylation of ammonia.

In 1995, the Baiker group reported the first example on the synthesis of methylamines from ammonia, H_2_ and CO_2_ through Cu/Al_2_O_3_ catalysts in a fixed-bed microreactor (Scheme 34) [134], but the efficiency was not high enough. Subsequently, they tested various types of metal–alumina catalysts (Cu, Ag, Pt, Ni, Co, Fe) to further improve the catalytic activities and selectivities [135,136,137]. The best amine production rates were obtained with Cu–Mg–Al mixed oxides at 280 °C, and over 79% selectivity of methylamine was obtained. 

During the past decade years, many research groups continuously carried out the study on heterogeneous catalysis (Cu [138,139], Pd [140,141], Pt [142,143], Au [144,145], Re [146]) on methylation of amines with CO_2_ and H_2_, and a significant progress had been made totally (Table 4). For example, Shi and co-workers described effective heterogeneous catalyst systems CuAlO_x_ [138] and Pd/CuZrO_x_ [140] for the methylation of amines with CO_2_ and H_2_ as sources for the methyl group, respectively. Primary and second aromatic and aliphatic amines were converted to methylamines under the identical reactions (170 °C, H_2_/CO_2_: 6–7/3 MPa). In the same year, Shimizu et al. [142] reported a Pt-MoOx/TiO_2_ catalyst in the methylation of secondary amines under solvent-free conditions (0.5 MPa CO_2_ and 5 MPa H_2_). Besides the good performance in the wide scope of secondary amines, the catalyst was reused in methylation of N-methyl aniline with CO_2_ and H_2_ for at least ten cycles with no significant loss in its activity. Notably, the total TON during the successive 10 runs reached 433 higher than TONs of the homogeneous Ru catalysts. Recently, they also studied TiO_2_-supported Re catalyst (Re/TiO_2_) promoted the *N*-methylation of amines by using H_2_ and CO_2_ [146]. Re/TiO_2_ efficiently catalyzed the reaction of various amines including substituted anilines, tetrahydroquinolines, aliphatic secondary amines in 66–99% yield.

**Scheme 35:**
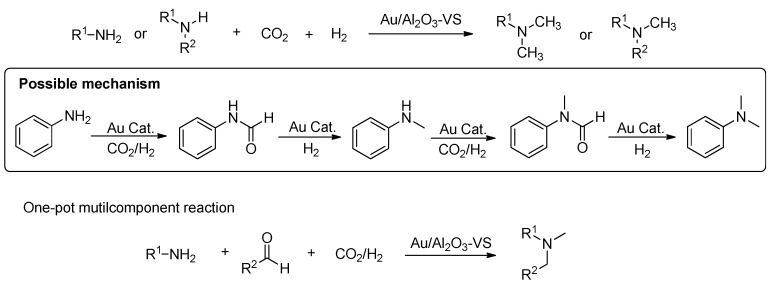
Au-catalyzed methylation of amine with CO_2_ and H_2._

In 2015, Su and Wang [144] demonstrated a supported Au catalyst (Au deposited on alumina) which was highly effective for the methylation of both aromatic and aliphatic amines using CO_2_/H_2_ to *N*-methylated products (Scheme 35). The average TOF based on surface Au atoms was 45 h^−1^ in the methylation of aniline with CO_2_ and H_2_. Moreover, in their work, a variety of amines including aromatic, aliphatic, secondary, and primary amines were demonstrated with good performance (40–99% yield). Notably, the catalytic scheme could make the one-pot method of primary amines, aldehydes, and CO_2_ with H_2_ to unsymmetrical tertiary amines. In addition, to extend the work, Du et al. revealed that with the decrease of the Au nanoparticle size from 8.3 to 1.8 nm, the TOF values for methylation of *N*-methylaniline with CO_2_/H_2_ increased [145].

**Scheme 36:**
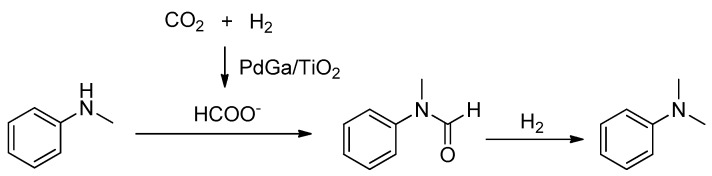
Reaction route of PdGa-catalyzed methylation of N-methylaniline [141].

Zhao et al. demonstrated that PdGa bimetallic alloy nanoparticles were highly dispersed on the TiO_2_ support, and the CO_2_ could be activated through the interaction between the electron-deficient Pd and Ga, which was confirmed by several detailed characterization studies (TEM, TPR, XPS, CO-adsorption IR and high-pressure in situ FTIR). The generation of a formic acid intermediate was proposed for the methylation (Scheme 36) [141]. As a result, 98% conversion and 94% selectivity for *N*-methylaniline was obtained under the identical conditions (180 °C, 5 MPa H_2_, 5 MPa CO_2_, 10 h).

**Scheme 37:**
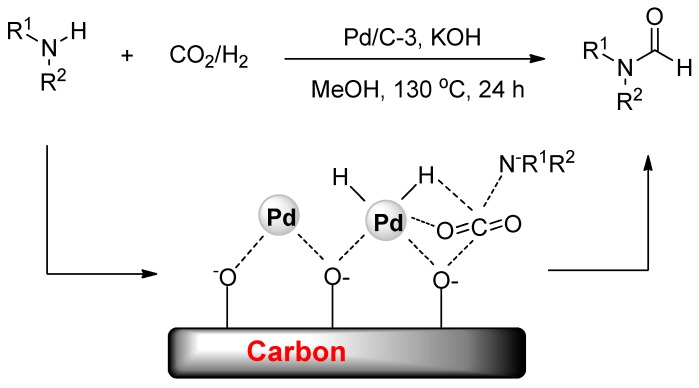
Heterogeneous palladium catalysis N-formylation of amines [147].

The reductive *N*-formylation of amines using CO_2_ and hydrogen is also a promising means of fixing CO_2_ into value-added chemicals, and heterogeneous catalysis schemes were also well developed recently (Table 5). Lately, the Shi group were the first to report a highly active supported palladium nanoparticle catalyst for the N-formylation of various aliphatic amines with CO_2_ and H_2_ (Scheme 37) [147]. During the preparation, Pd(NH_3_)_x_Cl_y_ was initially adsorbed onto the carbon support, and then the active nano-Pd particles were generated by *in situ* reduction. Additionally, the activity of the Pd/C catalysts can be tuned by -OH groups through modulating the hydrophilic/hydrophobic properties of the carbon surface which further potentially promote the adsorption of CO_2_ and amines near the Pd sites.

At the same time, a bimetallic Pd−Au catalyst (Pd/Au molar ratio of 1:1) prepared by depositing Pd−Au alloy nanoparticles (around 3.0 nm) on polyaniline-functionalized carbon nanotubes (PANI-CNT) was demonstrated the excellent catalytic activity for the *N*-formylation of pyrrolidine using CO_2_/H_2_ [148]. Ju et al. revealed that Pd atoms are the true active sites for the hydrogenation reaction, and the interaction between Pd atoms and Au atoms on bimetallic Pd−Au/PANI-CNT is beneficial to enhance the catalytic performance through changing the electronic properties of the formed bimetallic Pd−Au nanoparticles. In the work, a variety of aliphatic secondary amines showed excellent reactivity and gave high yield (67.2–98.3%). The aliphatic primary amines afforded a lower yield (up to 62.2%), and the tert-butylamine nearly performed no reactivity (trace product). Meanwhile, aniline was also not suitable under the reaction condition (0.1% yield).

Recently, a Pd nanoparticle (average size <2.0 nm) catalyst supported on N-doped carbons (NCs) was prepared by Liu group and applied for effective *N*-formylation of amines with CO_2_ and H_2_ in ethanol [149]. The investigation revealed that the interaction between the Pd nanoparticles and nitrogen in the NC support was responsible for the good performance of the catalyst. The catalyst was reused for three times, and the product yield decreased (from 93% to 81%). TEM observations indicated the reason may be the aggregation of Pd nanoparticle (from 2 to 3.5 nm) under the experimental conditions. In addition, a serial of aliphatic primary and secondary amines showed good reactivity and afforded the target products in high yields (51–99%). However, the aromatic amines such as aniline and *N*-methylaniline gave the corresponding products in low yield (11%, 11%) under the same conditions. 

## 4. Summary and Outlook

In conclusion, C-N bond formation reaction through CO_2_ as C_1_ source represents a big stride forward for the development of sustainable and green chemistry. In this work, we have summarized recent the advances of CO_2_ conversion to valuable chemicals from various N-contained substrates through metal or organocatalysis strategies. C-N bond formation has been achieved involving direct carboxylation/cyclization of CO_2_ with various N-contained substrates (such as propargyl amines, amino alcohols, allylic amines, allenic amines, etc.), sequential carboxylation and cyclization of propargyl alcohols, amines and CO_2_, as well as *N*-methylation/*N*-formylation of amine via CO_2_ hydrogenation. Among them, from the availability and the economic viability viewpoint, the multi-component cascade reaction of amines, CO_2_ and other nucleophilic candidates is especially receiving increased attention in recent years. Beside the advantages such as the thermodynamically favourable procedure, nontoxic and easily accessible material, the cascade approach was accomplished under mild reaction conditions with high efficiency. The insights on CO_2_ conversion through C-N bond formation reactions would be beneficial for the exploration of new strategies and directions on effective incorporation of CO_2_ into high value-added products.

Gratifyingly, encouraging advances on the development of novel strategies to construct organic molecules through C-N bond formation have been made, however, many problems and challenges still remain. Firstly, in most transformations involving CO_2_ conversion, the catalytic performance in transition metal systems is much better than that of organocatalysis strategies. Generally, the metal complexes easily forming active sites are more beneficial for activating the substrates such as alkene, alkyne and H_2_, etc. As a result, the catalytic efficiency and selectivity show much higher values, together with the lower catalytic loading, faster reaction rates, shorter times, and lower pressures and temperatures. Despite the superiority of metal complexes, their preparation processes are stricter (they are generally sensitive to water and air) and their stability is lower than that of organocatalysts. Also, the catalytic ability of organocatalysts has improved greatly. In addition, almost all of the metal-based catalysts were focused on the transition metals. Accordingly, the number of examples exploring main group metal-catalyzed transformations of CO_2_ is inadequate. Secondarily, the model products could be generally obtained in excellent yield and selectivity, but the examples of products being directly used as functional target molecules for medicines, natural products, or bioactive polymers, etc. are limited. Thus, the compatibility of catalytic methods in larger molecules bearing with much more complicated functional groups needs more exploration. Furthermore, for CO_2_ hydrogenation with amines, high yields and selectivities are achieved much easier using hydrosilanes than H_2_ as hydrogen source. However, the use of hydrosilanes will be lead to a large amount of siloxane by-products. Therefore, from the point of view of sustainable chemistry, the development of benign H_2_ process would have great significance and the exploration of robust methods for selective and effective catalysis is also essential despite being challenging. Besides, the efficiency of the noble transition metal-based homogenous schemes and metallic oxide-based heterogeneous catalytic systems must be further improved. Last but not least, it is challenging and imperative to discover more available methods and routes to transform CO_2_ into valuable chemicals with C-N bonds.

## Figures and Tables

**Table 1:** Silver-containing catalytic systems.

Catalyst (catalytic amount/mol%)	Solvent	T/°C	P/MPa	t/h	Yield/% ^a^	Ref.
Ag_2_CO_3_ (1.5), PPh_3_ (6)	CH_3_CN	30	0.1	16	68–98	[32]
Ag_2_WO_4_ (1), PPh_3_ (2)	-	50	0.5	12	30–97	[21]
Ag_2_O (2), PPh_3_ (8)	CH_3_CN	60	-	18	37–96 ^b^	[33]
Ag_2_CO_3_ (0.01), (*p*-MeOC_6_H_4_)_3_P (0.04)	CHCl_3_	25	2	96	30.5–93.2 ^c^	[34]
AgCl (5), Et_4_NCl (9)	CH_3_CN	60	0.1	24	29–98 ^b^	[35]

^a^ Isolated yield; ^b^ NMR yield; ^c^ gas chromatography (GC) yield.

**Table 2:** One-pot three-component reactions of propargylic alcohols, CO_2_ and 2-aminoethanols.

Catalyst (Catalytic Amount/mol%)	Solvent	T/°C	P/MPa	t/h	Yield(2-oxazolidinones)/% ^a,b^	Ref.
Ag_2_CO_3_ (5), Xantphos (10)	CHCl_3_	60	1	18–48	33–92	[69]
Ag_2_O (5), TMG (30)	CH_3_CN	80	1	12	24–99	[70]
CuI (5), 1,10-phen (5), *t*-BuOK (10)	-	80	0.5	12	24–97	[66]
[TBDH][TFE] (15)	-	80	0.1	12	24–99	[71]

^a^ NMR yield.; ^b^ Isolated yield.

**Table 3:** Metal-catalyzed carbonylation of amines and CO_2._

Catalyst	Solvent	T/°C	P/MPa	t/h	Yield/% ^a^	Ref.
CeO_2_	NMP	130	5	24/48	<1–86	[92]
Y_2_(C_2_O_4_)_3_^b^	NMP	150	CO_2_(2)/N_2_(1)	24	62.43	[93]
Y_0.08_Zr_0.92_O_1.96_^b^	NMP	160	CO_2_(2)/N_2_(1)	12–24	47.14–80.60	[94]
In(N(SiMe_3_)_2_)Cl_2_·(THF)_n_, pyridine	-	110	0.3	3–90	-	[95]

^a^ GC yield; ^b^ 4A zeolite as dehydrant.

**Table 4:**
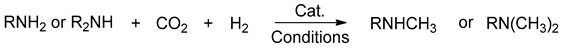
Methylation of selective amines with CO_2_ and H_2_.

Entry	Sub.	Cat.	P_CO2_ /MPa	P_H2_/MPa	Temp./°C	T/h	Sol.	Prod.	Conv./% ^a,b^	Yield/% ^a,b^	Sel./% ^a,b^	Ref.
1	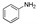	CuAlO_x_	3	6	160	24	hexane	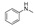	NA	86	NA	[138]
2	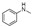	CuAlO_x_	3	7	160	24	hexane	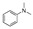	NA	86	NA	[138]
3	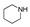	CuAlOx	3	7	160	24	hexane	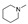	NA	87	NA	[138]
16	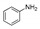	Cu/CeO_2_	1	7	160	4	toluene	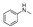	28	27	95	[139]
6	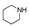	Pd/CuZrO_x_	1	2.5	150	30	octane	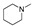	NA	91	NA	[140]
7	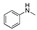	Pd/CuZrO_x_	1	2.5	150	30	octane	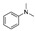	NA	71	NA	[140]
8	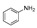	Pd/CuZrO_x_	1	2.5	150	30	octane	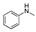	NA	75	NA	[140]
15	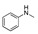	PdGa/TiO_2_	5	5	180	10	octane	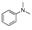	98	92	94	[141]
9	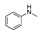	Pt-MoO_x_/TiO_2_	0.5	5	200	24	neat	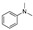	85	85	100	[142]
10	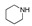	Pt-MoO_x_/TiO_2_	0.5	5	200	24	neat	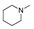	100	63	63	[142]
4	NH_4_HCO_3_	Pt-MoO_x_/TiO_2_	1	5	250	24	neat	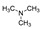	NA	65	NA	[142]
5	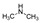	Pt-MoO_x_/TiO_2_	1	5	250	24	THF	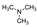	NA	56	NA	[143]
12	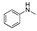	Au/Al_2_O_3_-VS	2	6	140	7	hexane	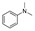	NA	92a	NA	[144]
13	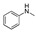	Au/Al_2_O_3_-VS (Au: 0.5 mol%)	2	6	140	7	hexane	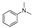	NA	96	NA	[144]
14	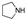	Au/Al_2_O_3_-VS (Au 0.5 mol%)	2	6	140	7	hexane	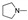	NA	99	NA	[144]
11	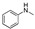	Au/Al_2_O_3_ (Au Size 2.0 nm, Au 0.73 wt%)	1	3	140	5	cyclohexane	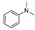	76	>75	>99	[145]
17	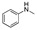	Re/TiO_2_	1	5	200	24	octane	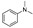	NA	98	NA	[146]
18	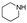	Re/TiO_2_	1	5	200	24	octane	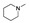	NA	99	NA	[146]

^a^ GC conversion and yield based on amine. ^b^ Not available (NA).

**Table 5:**
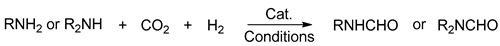
N-Formylation of typical amines with CO_2_/H_2_.

Entry	Sub.	Cat.	P_CO2_ /MPa	P_H2_/MPa	Temp./^o^C	T/h	Sol.	Prod.	Conv./% ^a,b^	Yield/% ^a^	Ref.
1	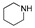	Pd/C	1	3	105	24	MeOH	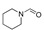	NA	93	[147]
2	*^n^*BuNH_2_	Pd/C	1	3	105	24	MeOH	*^n^*BuNHCHO	NA	77	[147]
3	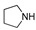	Pd−Au/PANI-CNT	3.5	3.5	125	48	1,4-dioxane	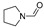	>99.9	98.3	[148]
4	*^n^*BuNH_2_	Pd−Au/PANI-CNT	3.5	3.5	125	48	1,4-dioxane	*^n^*BuNHCHO	63.1	62.2	[148]
5	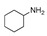	Pd−Au/PANI-CNT	3.5	3.5	125	48	1,4-dioxane	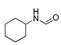	4.1	3.4	[148]
6	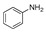	Pd−Au/PANI-CNT	3.5	3.5	125	48	1,4-dioxane	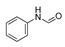	0.2	0.1	[148]
7	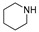	Pd/NC	3	4	130	24	ethanol	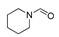	NA	94	[148]
8	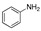	Pd/NC	3	4	130	24	ethanol	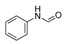	NA	11	[148]

^a^ GC conversion and yield based on amine. ^b^ Not available (NA).

## Abbreviations

Carbon dioxide (CO_2_); ionic liquids (ILs); primitive-cubic (pcu); metal-organic framework (MOF); nuclear magnetic resonance (NMR); gas chromatography (GC); high-resolution mass spectrometry (HRMS); *N*-heterocyclic carbene (NHC); nanoparticles (NPs); diphosphine-borane (DPB); 1,8-diazabicyclo[5.4.0]undec-7-ene (DBU); dimethyl sulfoxide (DMSO); density functional theory (DFT); polyoxometalate (POM); 1,5,7-triazabicyclo[4.4.0]dec-5-ene (TBD); tetra-*n*-butylammonium (TBA); cyanuric acid (H_3_CYA); triethanolamine (TEOA); *p*-toluenesulfonyl chloride (TsCl); 1,5,7-triazabicylo[4.4.0]dec-5-ene ([TBDH][TFE]); *N*-methyl-2-pirrolidinone (NMP); 3-amino-1*H*-1,2,4-triazole (ATriz); 4,7,10-trioxa-1,13- tridecanediamine (TOTDDA); attenuated total reflectance fourier transform infrared spectroscopy (ATR-FTIR); polymethylhydrosiloxane (PMHS); butylammonium fluoride (TBAF); turnover frequency (TOF); polyaniline-functionalized carbon nanotubes (PANI-CNT); *N*-doped carbons (NCs)
